# Identification of Somatic Mutations in Papanicolaou Smear DNA and Plasma Circulating Cell-Free DNA for Detection of Endometrial and Epithelial Ovarian Cancers: A Pilot Study

**DOI:** 10.3389/fonc.2020.582546

**Published:** 2020-12-14

**Authors:** Xuan Jiang, Weihua Li, Jiaxin Yang, Shuzhen Wang, Dongyan Cao, Mei Yu, Keng Shen, Jian Bai, Yang Gao

**Affiliations:** ^1^ Department of Obstetrics and Gynecology, Peking Union Medical College Hospital, Chinese Academy of Medical Science & Peking Union Medical College, Beijing, China; ^2^ Department of Obstetrics and Gynecology, Beijing Chao-yang Hospital, Capital Medical University, Beijing, China; ^3^ Berry Oncology Corporation, Beijing, China

**Keywords:** somatic mutation, endometrial cancer, epithelial ovarian cancer, circulating cell-free DNA, Papanicolaou smear

## Abstract

**Objectives:**

The aim of this study was to identify tumor-derived DNA from Papanicolaou (Pap) smear and plasma specimens collected from patients with endometrial cancer or atypical hyperplasia (EC/AH) or epithelial ovarian cancer (OC).

**Methods:**

Tumor tissues, peripheral blood, and Pap smear samples were collected from patients with EC/AH and patients with epithelial OC. Somatic mutations of tumor specimens in EC/AH and OC were examined by whole-exome sequencing using a 127-driver gene panel from The Cancer Genome Atlas (TCGA). A nine-gene EC/AH panel and an eight-gene OC panel were established based on the identified significantly mutated genes in the EC/AH and OC tumor specimens. Circulating single-molecule amplification and resequencing technology (cSMART) was applied to evaluate somatic mutations in Pap smear DNA and plasma circulating cell-free DNA (ccfDNA) using the EC/AH and OC gene panels.

**Results:**

In EC/AH group, there existed 22 tumors and 14 of the 22 tumors contributed hot spot mutations for the EC/AH nine-gene panel. In the Pap smear subgroup, all 21 Pap smears tested positive. Nine out of 11 (81.8%) identified the same gene mutations with their matched tumors and the remaining 10 Pap smears all tested positive. In the plasma subgroup, 10 out of 26 (38.5%) plasmas tested positive. One out of 13 (7.7%) identified the same gene mutation with its matched tumor and 5 out of the remaining 13 plasmas (38.5%) tested positive. In OC group, there existed 17 tumors and 16 of the 17 tumors contributed hot spot mutations for the OC eight-gene panel. In the Pap smear subgroup, all 11 Pap smears tested positive. Five out of 10 (50.0%) identified the same gene mutations with their matched tumors and the remaining one Pap smear also tested positive. In the plasma subgroup, all 22 plasmas tested positive. Ten out of 14 (71.4%) identified the same gene mutation with their matched tumors and the remaining 4 plasmas all tested positive.

**Conclusions:**

Tumor-derived DNA can be detected in Pap smears and plasmas from patients with EC/AH or epithelial OC. Using a small gene-panel, early detection of EC/AH and OC might be promising. However, the value of plasma ccfDNA for EC/AH requires further investigation.

## Introduction

Among the tumors of the female reproductive tract, known as Mullerian duct tumors, epithelial ovarian cancer (OC) comprises the most intractable tumors with high rates. Epithelial OC is the leading cause of gynecologic cancer-related deaths in women, primarily because most patients present with advanced-stage disease. The 5-year survival rate remains at a low level (1, 2). Moreover, in the last 20 years, the incidence rate of endometrial cancer (EC) in the world has increased by 125%, and the death rate has increased by 65.2% (3, 4). Despite improved diagnosis and treatment methods, up to 30% of EC patients are primarily diagnosed with stage III or IV EC and have poor outcomes. More techniques are needed for detecting these tumors at an early stage and helping improve the survival of these patients.

Recently, researchers have found that all cancers are caused by somatic mutations (5–7). The mutation process of cells reflects what has occurred in the cells and helps us to understand the biological processes generating these mutations. Thus, by studying the mutation process, it is expected that clues about the early changes in cancer cells and even the characteristics of cell mutations in the precancerous stage will be found. Researchers have found nearly 140 gene mutations that promote or initiate tumorigenesis. These gene mutations are known as “driver gene mutations.” In contrast, more than 99.9% of tumor gene mutations are insignificant passenger gene mutations, and their occurrence marks the end of well-regulated cell proliferation. When passenger mutations occur, apoptosis is automatically initiated. However, upon TP53 gene mutation, the cell will automatically acquiesce to the mutation and become cancerous (8). In general, tumor occurrence requires two to eight driver gene mutations, as well as numerous passenger gene mutations (5, 8). Most of these mutations are single-base substitutions, whereas the remainder are small deletions or insertions of one or a few bases (5, 8). The Cancer Genome Atlas (TCGA) identifies 127 driver genes significantly mutated in various tumors, based on the results of whole-genome and whole-exome sequencing of more than 3,000 tumor specimens covering 12 histological types. The “pan-cancer panel” is composed of 7,816 probes covering 800 kb of the human genome that was designed to detect significantly mutated genes among the 127 driver genes in tumor tissues (9, 10).

It is well-known that the incidence and mortality of cervical cancer have been tremendously reduced since the introduction of the Papanicolaou (Pap) smear for cytology and human papillomavirus (HPV) testing as routine screening for cervical cancer (11). The routinely collected cells can also be exploited to detect somatic mutations present in rare tumor cells that accumulate in the cervix once shed from EC or OC. By analyzing the cells shed from the cervix, the state of the female reproductive organs, such as the ovaries, fallopian tubes, endometrium, and cervix, can be well indicated. Endocervical DNA or intrauterine DNA can be obtained through vaginal tampons (12), intrauterine lavage (13, 14), Pap smear, and Tao Brush (11, 15) to determine the presence of malignant tumor cells in the reproductive tract. Circulating tumor DNA is a type of cell-free DNA that accounts for a small part of circulating cell-free DNA (ccfDNA). It is usually released from tumor tissues *in vivo* and harbors tumor-specific DNA sequences, which is a good indicator of tumor status *in vivo*. ccfDNA carrying tumor-specific somatic mutations can be thought of as tumor-derived DNA (16, 17).

As TCGA data analysis of EC and high grade serous OC (HGSOC) has yielded clinically significant results, we presume that liquid biopsy technology may be used to detect Mullerian cancers at an early stage. Kinde et al. has thoroughly studied the possible role of Pap smear DNA in detecting EC or OC and our study has been inspired by their first article (11). Basically, it has been confirmed that tumor cells shed from EC or OC can be collected from Pap smear in the cervix, since the identical somatic mutation from tumor tissues of EC or OC can be detected in Pap smear cells. It is critical to confirm the somatic mutations from Pap smear cells come from the malignancy of female reproductive tract. However, the study of Kinde et al. did not set the self-control of leukocytes and they did not systematically and comprehensively evaluate the somatic mutations in tumor tissues. Through the establishment of self-control of leukocytes, the somatic mutations unique to tumor tissues were determined. And the existence of these unique mutations were simultaneously validated in Pap smear DNA or ccfDNA. This can very well prove that these somatic mutations detected in Pap smear or plasma were derived from tumor tissues *in vivo*. However, because of tumor heterogeneity and the limitation of tumor sampling, somatic mutations detected in Pap smear or plasma, which were not the same with those identified in tumor tissues, should not necessarily be considered as non-neoplastic origin. It is important to eliminate the cases of false positive and also keep the cases of false negative. The multiple gene-panel may be an efficient way to solve this problem. The establishment of gene-panel in this study was derived from the whole exome sequencing of tumor tissues through analysis of 127 driver genes and thus the panel was more accurate and targeted. The objective of this study was to identify tumor-derived DNA from Pap smear and plasma specimens collected from patients with EC or epithelial OC through analysis of somatic mutations in driver genes.

## Materials and Methods

### Patient Selection

Between November 2014 and June 2015, patients undergoing treatment for newly diagnosed epithelial ovarian, fallopian tube or primary peritoneal cancer, or endometrial cancer or atypical endometrial hyperplasia were enrolled in a study approved by the Ethics Committee of Peking Union Medical College Hospital (PUMCH) and provided written informed consent. Patients who were highly suspected as having invasive cervical cancer, a precancerous lesion, or human papillomavirus (HPV) infection were excluded.

### Sample Collection and Processing

Fresh tumor tissue was collected from the primary carcinoma site during the operation, and shed cervical cells were collected using Pap smears and a liquid-based method. Peripheral blood from patients was collected in a CF tube prepared with a special material containing a protective agent. The blood was allowed to stand at room temperature (15–25°C) for 1 h, and the protective agent was fully reacted; the sample was then stored at 4°C. Blood samples were processed within 24 h of collection. The blood was separated by centrifugation at 1,600 × g for 10 min at 4°C, and the plasma was again centrifuged at 16,000 × g for 10 min to remove impurities to obtain fresh plasma.

### Extraction and Quantification of Genomic DNA (gDNA) and ccfDNA

A mini-spin column was utilized to extract gDNA from tumor tissues and sloughed-off cervical cells (Qiagen DNeasy Blood & Tissue Kit). Samples were first lysed using proteinase K. Buffering conditions were adjusted to provide optimal DNA-binding conditions, and the lysate was loaded onto a DNeasy Mini spin column. During centrifugation, the DNA is selectively bound to the DNeasy membrane as contaminants pass through. The remaining contaminants and enzyme inhibitors are removed in two efficient wash steps, and the DNA is then eluted in water or buffer.

Peripheral leukocyte gDNA and ccfDNA were extracted using magnetic beads. Leukocyte gDNA extraction was carried out using an AxyPrep Mag Tissue-Blood gDNA Kit (Axygen), which begins by the addition of lysis buffer and proteinase K to rupture cell membranes and digest protein. gDNA is then immobilized on magnetic particles by the addition of a magnetic binding reagent. This differential binding allows the gDNA to be easily separated from contaminants using a magnetic field. Contaminants can then be rinsed away using a simple washing procedure. ccfDNA extraction was performed with a MagMAX Cell-Free DNA Isolation Kit. The plasma samples were centrifuged at 16,000 × g for 10 min at 4°C and treated with proteinase K; the ccfDNA was then bound to beads. Finally, the ccfDNA was washed with wash solution and 80% ethanol and eluted with elution solution.

All DNA samples were purified, and quantified with a Qubit Fluorometer and the Qubit dsDNA HS Assay Kit (Invitrogen). Samples with tumor gDNA (≥50 ng), peripheral leukocyte gDNA (≥50 ng), Pap smear DNA (≥250 ng), or ccfDNA (≥20 ng) met the standard for subsequent experiments. All gDNA, except ccfDNA, was required to be fragmented.

### Next-Generation Sequencing (NGS) Library Construction

Tumor gDNA and leukocyte gDNA were ligated to universal sequencing adaptors and amplified by PCR. The preliminary DNA libraries were subjected to hybridization and target capture using Pan-Cancer Panel [xGen Lockdown Probes and Panels (IDT)], which consists of 7,816 probes and spans 800 kb of the human genome. Blocking oligonucleotides were added to the gDNA library fragments prepared, binding to Nextera adapter sequences on a designated strand to reduce nonspecific binding and improve the number of target reads. The hybridized library fragments were incubated with streptavidin-coated magnetic beads, and the bead-bound targets were isolated with a magnet. Libraries were then reamplified by PCR and subjected to paired-end sequencing using the Illumina HiSeq 2500 platform.

After quality control, the primary sequencing data were aligned/mapped to the human genome reference sequence, and redundant data were eliminated/filtered. Sequencing errors are frequent on all current sequencing platforms, and they are difficult to separate from true low-frequency single-nucleotide variants. Single-nucleotide errors can result from target enrichment, library preparation, and base calling. To overcome these limitations, a comparative sequencing strategy was adopted, whereby the same genomic region was compared between a tumor sample and a self-leukocyte control sample using a customized statistical algorithm (18). Thus, to eliminate self-benign mutations and some sequencing errors, the sequencing data for tumor tissues were compared with those of matched leukocytes. Finally, the somatic mutated allelic variants were identified and analyzed. The proportion of somatic mutated allelic variants in each tumor specimen was counted, and specimens with an extremely large number of mutated allelic variants (microsatellite instability, MSI) were excluded. Mutated genes with a frequency greater than two in tumor specimens were selected as candidate genes. Significantly mutated allelic variants in candidate genes were ultimately identified *via* combination with the mutant allele fraction and functional prediction using online software.

### cSMART

Tumor-derived DNA only accounts for a small part of the Pap smear DNA or ccfDNA; therefore, the sensitivity of the detection technology used must be very high, and its consistency must also be high to ensure the accuracy and reliability of the data obtained. In this study, we adopted circulating single-molecule amplification and resequencing technology (cSMART) to build a library. cSMART can result in libraries with good specificity and sequencing depth, and as reverse primers can be designed to be very close to the target region (hot spot mutation), most template DNA containing the target region can be used for polymerase chain reaction (PCR) amplification. This method effectively uses template DNA molecules to build a library. The detection sensitivity of the target DNA sequence for cSMART analysis was 1/10,000, and the sequencing coverage of all hotspots was 10,000×. The target DNA molecule templates captured in each hotspot numbered more than 100.

Uniquely barcoded single allelic molecules in the ccfDNA and Pap smear DNA samples were counted to detect and quantitate the level of mutations using a multiplex cSMART assay. Adaptors were added to end-modified fragmented DNA and libraries generated by PCR amplification. Library molecules were uniquely barcoded and then circularized by ligation with an oligonucleotide containing a 4-bp degenerate sequence. A pair of “back-to-back” primers were designed upstream and downstream of each mutation locus, and a total of 27 and 19 bidirectional back-to-back targeting primer pairs were designed to simultaneously detect 38 and 29 hot spot mutations in a nine-gene panel for EC/AH (*PTEN*, *PIK3CA*, *TP53*, *ARID1A*, *CTNNB1*, *ARHGAP35*, *SPOP*, *SOX7*, and *PIK3R1*) and an eight-gene panel for OC (*TP53*, *ARID1A*, *LRRK2*, *BRCA1*, *ARHGAP35*, *EGFR*, *RAD21*, and *PIK3CA*) (the sequences of the primer pairs and gene panels for EC/AH or OC for cSMART analysis are listed in **Supplementary Material 1**). Following amplification of the targeted alleles by inverse PCR, paired-end sequencing was performed using the Illumina HiSeq 2500 platform. Based on overlapping paired-end sequences of each molecule, single alleles were reconstructed using FASTQ-join software (http://code.google.com/p/ea-utils). A minimum of 100 unique allelic molecules were counted. The mutation ratio was calculated as the number of mutant alleles/total alleles.

## Results

### Patients and Samples

During the study period (November 2014–June 2015), there were 70 patients with suspected epithelial ovarian, fallopian tube or primary peritoneal cancer (OC), or endometrial cancer or atypical endometrial hyperplasia (EC or AH) at our clinic, who were fully informed of the risks and benefits of participating the study. At the beginning of enrollment, patients should be informed that all samples should be taken without prejudice to diagnosis and treatment. For example, tumor tissue sampling should ensure that enough tumor tissue is sent for pathological examination. The Pap smear sampling was made from the remaining cells after the patient had undergone a cervical cytology examination. Therefore, some endometrial cancer or atypical hyperplasia, with small lesions or even invisible to the naked eye, were difficult to obtain tumor tissues.

Sixty-five of the 70 patients agreed to participate in the study if they had pathologically confirmed epithelial OC, EC, or AH. Fresh tumor specimens were collected from patients during surgery. Peripheral blood and Pap smear samples were collected from patients before surgery. Ultimately, 55 of the 65 patients had pathologically confirmed epithelial OC, EC, or atypical hyperplasia. Four of the 55 patients dropped out of the study and 51 patients were enrolled in the study.

The clinical and pathological variables and prognoses of the 51 patients (32 EC or AH and 19 OC) are shown in **Table 1**. In EC or AH group, the median age was 47 years. Eight (25.0%) patients were pathologically diagnosed with atypical endometrial hyperplasia, 21 (65.6%) with endometrioid carcinoma, 1 (3.1%) with clear cell carcinoma, 1 (3.1%) with endometrioid mixed with clear cell carcinoma, and 1 (3.1%) with endometrioid mixed with serous carcinoma. Most ECs (83.3%) were grade 1 or grade 2, and most (83.3%) were staged as IA. All patients were alive with no evidence of tumors. In OC group, the median age was 52 years. Sixteen (84.2%) patients were pathologically diagnosed with HGSOC, 2 (10.5%) with ovarian clear cell carcinoma, and 1 (5.3%) with endometrioid OC. Most ovarian tumors (94.7%) were grade 3, and most (68.4%) were staged as IIIC. Five (26.3%) patients survived, and 11 (57.9%) died of the tumor.

**Table 1:** The clinicopathologic characteristics and prognosis of patients.

	EC/AH (n = 32)		OC (n = 19)	
Age (Median, min-max, years)	47 (26–84)		52 (34–82)		
Histology	AH	8 (25.0%)	HGSC	16 (84.2%)	
	Endometrioid	21 (65.6%)	CCC	2 (10.5%)	
	Endometrioid + CCC	1 (3.1%)	Endometrioid	1 (5.3%)	
	Endometrioid + serous	1 (3.1%)			
	CCC	1 (3.1%)			
Grade	Grade 1	12 (50.0%)	Grade 2	1 (5.3%)	
	Grade 2	8 (33.3%)	Grade 3	18 (94.7%)	
	Grade 3	4 (16.7%)			
FIGO stage	IA	20 (83.3%)	IC3	1 (5.3%)	
	IB	1 (4.2%)	IIB	2 (10.5%)	
	IIIB	1 (4.2%)	IIIB	1 (5.3%)	
	IIIC1	1 (4.2%)	IIIC	13 (68.4%)	
	IV	1 (4.2%)	IVA	1 (5.3%)	
			IVB	1 (5.3%)	
Outcomes	NED	32 (100%)	NED	3 (15.8%)	
	AWD	0 (0%)	AWD	5 (26.3%)	
	DOD	0 (0%)	DOD	11 (57.9%)	

NED, no evidence of disease; AWD, alive with disease; DOD, die of disease; AH, atypical hyperplasia; CCC, clear cell ovarian cancer; HGSC, high grade serous ovarian cancer.

### Tumor Tissues and Matched Leukocytes

In EC/AH group, tumor tissues were not available in seven patients due to small or invisible lesions. In OC group, tumor tissue was not available in one patient. Therefore, only 43 tumors were collected in 51 patients. Tumor gDNA and matched leukocyte gDNA were subjected to whole-exome sequencing of 127 driver genes. Three tumors were tested numbers of somatic mutations and much higher than the other tumors. These tumors were considered as “microsatellite instability” and were excluded. And the leukocyte testing was failed in one OC patient. Thus 39 patients with matched tumors and leukocytes (22 EC or AH and 17 OC) were included in the study.

### Pap Smears

The main problem with Pap smear sampling was that the amount of DNA extracted did not meet the requirements of detection. In EC/AH group, 12 Pap smear specimens did not meet the standard of the extracted Pap smear DNA ≥250 ng. Besides, one AH patient was repeatedly sampled before the surgery. In OC group, eight Pap smear specimens did not meet the standard of the extracted Pap smear DNA ≥250 ng. Eventually, 32 Pap smear specimens (21 from 20 EC/AH patients and 11 from 11 OC patients) were included in the study. And the incidence of qualified Pap smear specimens was 62.5% (20/32) in EC/AH group and 57.9% (11/19) in OC group, respectively.

### Plasmas

**Figure 1:**
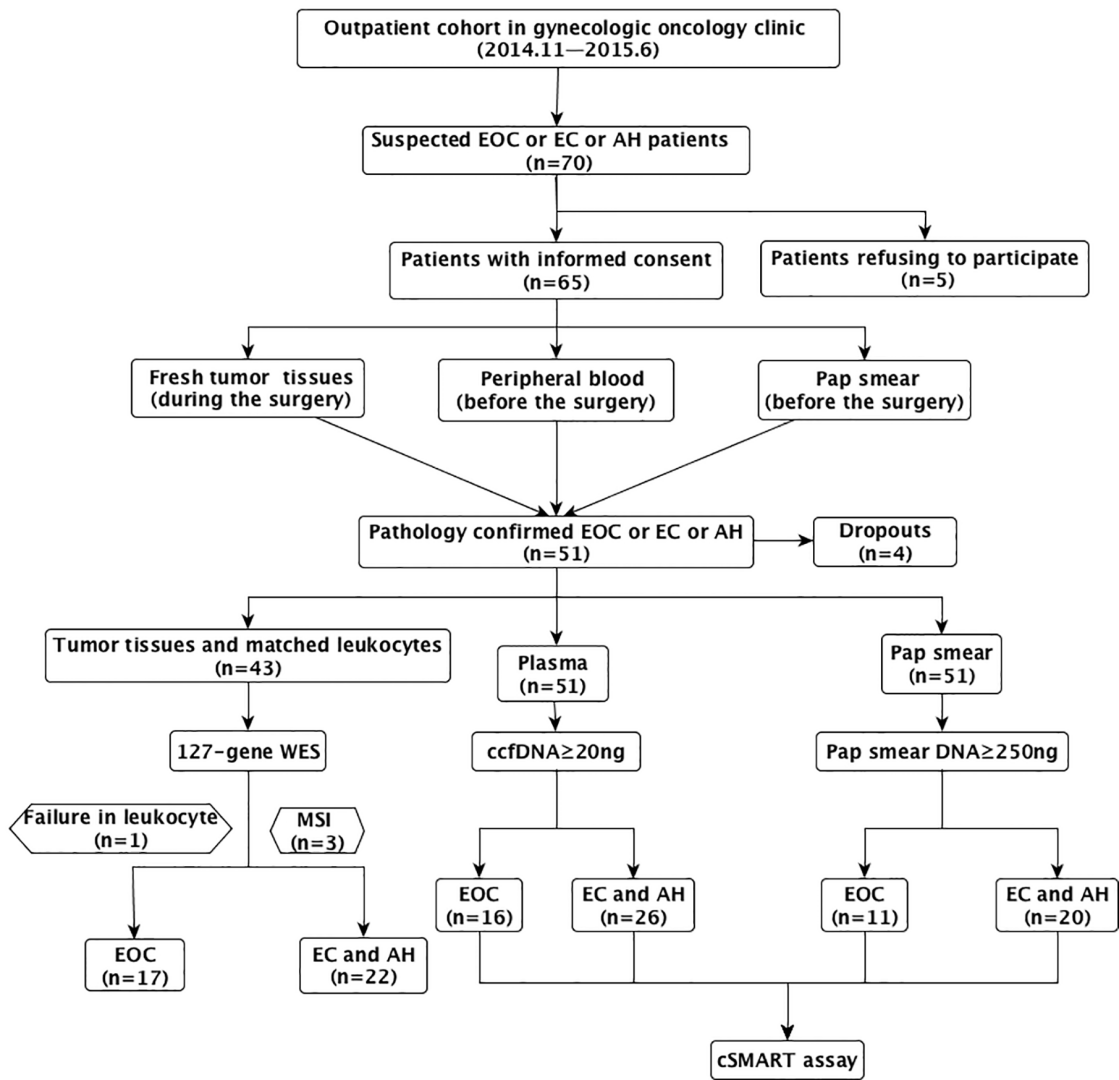
The selection process and patient exclusion criteria.

The main problem with plasma sampling was the high incidence of hemolysis, which made a low incidence of qualified ccfDNA. In EC/AH group, six plasma specimens did not meet the standard of the extracted ccfDNA ≥20 ng. In OC group, three plasma specimens did not meet the standard of the extracted ccfDNA ≥20 ng. Besides, three patients were sampled for three times before the surgery. Eventually, 48 plasma specimens (26 from 26 EC/AH patients and 22 from 16 OC patients) were included in the study. And the incidence of qualified plasma specimens was 81.3% (26/32) in EC/AH group and 84.2% (16/19) in OC group, respectively. The selection process and exclusion criteria are summarized in **Figure 1**.

### Spectrum of Mutations in Tumor Tissues

We evaluated the quality of the sequencing data. The average covering depth for target areas in tumor specimens was 660.2×; the depth was 298.7× for matched leukocytes. The average coverage was 99.9%, and 97.8% of the target areas were sequenced more than 10×. The quality and amount of the sequencing data met the standard of somatic mutation analysis (see details in **Supplementary Material 1**).

### EC/AH Tumors

By 127-gene whole-exome sequencing, 90 mutations involving 34 genes (26.8%, 34/127) were detected in the 22 EC/AH tumor specimens. The median numbers of mutated driver genes, mutated gene regions, and mutant allele fractions in EC/AH tumor specimens were 3 (0–13), 4 (0–16), and 16.8% (0–48.4%), respectively. PTEN was the most frequently mutated gene among the specimens (59.09%, 13/22), followed by PIK3CA (50.0%, 11/22), CTNNB1 (36.36%, 8/22), ARID1A (27.27%, 6/22), and TP53 (27.27%, 6/22). We divided the EC/AH patients into two groups: 1) patients with high-grade tumors, serous or clear cell carcinomas (the Poor group) and 2) patients with low-grade tumors or atypical hyperplasia (the Good group). The most frequently mutated gene in the Poor group was TP53 (71.43%, 5/7) and that the most frequently mutated gene in the Good group was PTEN (73.33%, 11/15), followed by PIK3CA (9/15, 60%), CTNNB1 (53.33%, 8/15), and ARID1A (26.67%, 4/15). In addition, the frequencies of mutated genes between the two groups were compared and some of the mutated genes were distributed significantly differently, such as TP53 and CTNNB1. Six of 22 (27.3%) EC/AH patients carried the TP53 nonsynonymous mutation. Among these mutations, five occurred in the Poor group but only one in the Good group, with a significant difference [5/7 (Poor) *versus* 1/15 (Good), p = 0.0043]. Eight of 22 (36.4%) EC/AH patients carried the CTNNB1 nonsynonymous mutation, all of which occurred in the Good group, with a significant difference [0/7 (poor) *versus* 8/15 (good), p = 0.020]. Moreover, the PTEN or PIK3CA gene mutation frequency was also different between the two groups, with a slightly higher frequency in the Good group than in the Poor group; however, the difference was not statistically significant. See in **Table 2**.

**Table 2:** The difference of somatic mutational gene frequency between Good and Poor EC patients.

GeneSymbol	Total prevalence	POOR group	GOOD group	P value
PTEN	13/22	2/7	11/15	0.064
PIK3CA	11/22	2/7	9/15	0.18
CTNNB1	8/22	0/7	8/15	0.020*
ARID1A	6/22	2/7	4/15	0.65
TP53	6/22	5/7	1/15	0.0043*
SOX17	3/22	2/7	1/15	0.23

* p < 0.05.

### OC Tumors

By 127-gene whole-exome sequencing, 71 mutations involving 41 genes (32.3%, 41/127) were detected in the 17 OC tumor specimens. The median numbers of mutated driver genes, mutated gene regions, and mutant allele fractions in OC tumor specimens were 4 (1–14), 4 (1–17), and 21.0% (3.0–65.5%), respectively. TP53 was the most frequently mutated gene (94.12%, 16/17), followed by ARID1A (23.53%, 4/17), and EPPK1 (23.53%, 4/17).

**Figure 2:**
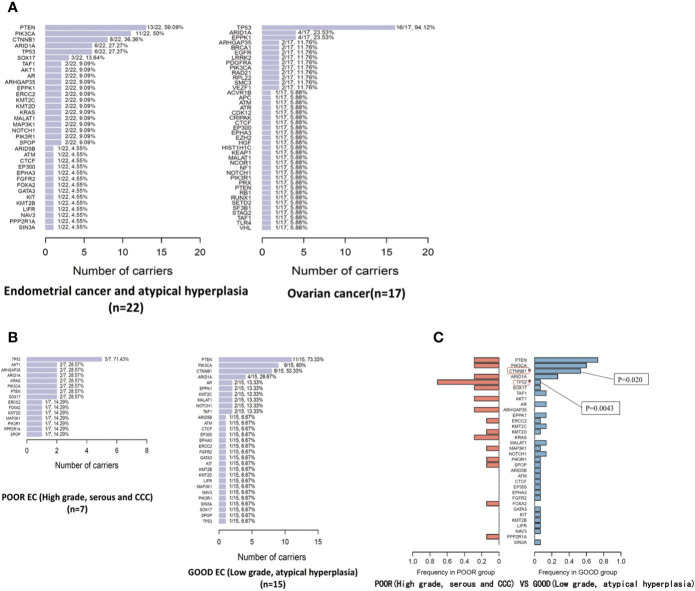
The mutated genes and mutation frequency in EC/OC tumor specimens **(A)** and in tumor specimens of Good/Poor EC **(B)**; comparison of mutated genes and mutation frequency in tumor specimens of Good/Poor EC **(C)**.

**Figure 3:**
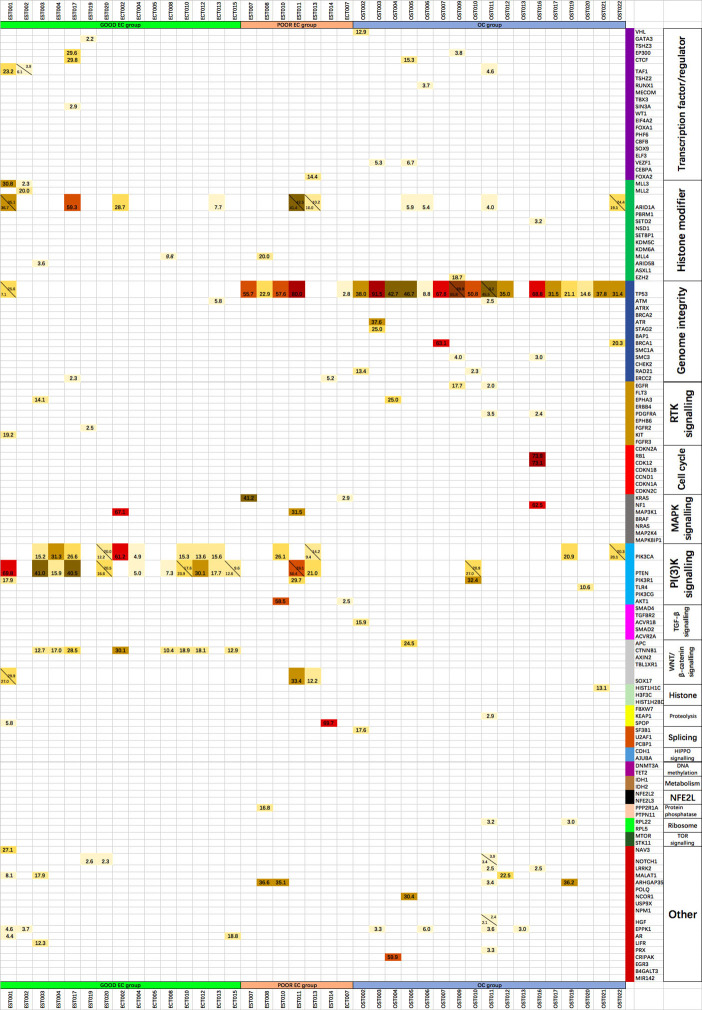
The mutant allele fractions of the 127 driver genes of 20 cellular processes in each tumor specimen, with the alleles in the same gene listed at the same grid separated by the diagonal.

The somatic mutated genes and frequency in EC/AH and OC tumor specimens are summarized in **Figure 2**. The mutated genes in each tumor specimen are provided in **Table 3** and **Figure 3**. ARID1A was commonly mutated across both EC/AH and OC tumors and TP53 across OC and Poor EC tumors; PIK3CA and PTEN mutations occurred across EC/AH tumors, especially Good EC/AH tumors, and CTNNB1 was mutated across Good EC/AH tumors but not in Poor EC/AH or OC tumors.

**Table 3:** The somatic mutations of endometrial and ovarian carcinoma.

A. Endometrial Carcinoma				
Arm	Sample	*Mutational genes*	MAF	Pathways involved
Good	EST001	*ARID1A, KMT2C/MLL3, SOX17, PTEN, TP53, TAF1, KIT, MALAT1, TP53, SPOP, AR, ARID1A, NAV3, PIK3R1, EPPK1, SOX17*	24.4%	Transcription factor/regulator; Histone modifier; Genome integrity; RTK signaling; PI(3)K signaling; WNT/β-catenin signaling; Proteolysis; Other
Good	EST002	*KMT2B/MLL2, TAF1, KMT2C/MLL3, EPPK1, TAF1*	3.9%	Transcription factor/regulator; Histone modifier; Other
Good	EST003	*PTEN, CTNNB1, EPHA3, PIK3CA, LIFR, ARID5B, MALAT1*	14.1%	Histone modifier; RTK signaling; PI(3)K signaling; WNT/β-catenin signaling; Other
Good	EST004	*PIK3CA, CTNNB1, PTEN*	17%	PI(3)K signaling; WNT/β-catenin signaling
Good	EST017	*ARID1A, CTNNB1, PIK3CA, PTEN, SIN3A, CTCF, ERCC2, EP300*	29.1%	Transcription factor/regulator; Histone modifier; Genome integrity; PI(3)K signaling; WNT/β-catenin signaling
Good	EST019	*FGFR2, GATA3, NOTCH1*	2.5%	Transcription factor/regulator; RTK signaling; Other
Good	EST020	*PIK3CA, PTEN, PIK3CA, PTEN, NOTCH1*	16.6%	PI(3)K signaling; Other
Good	ECT002	*ARID1A, CTNNB1, PIK3CA, MAP3K1*	45.7%	Histone modifier; MAPK signaling; PI(3)K signaling; WNT/β-catenin signaling
Good	ECT004	*PIK3CA, PTEN*	5.0%	PI(3)K signaling
Good	ECT005	*—*	*—*	*—*
Good	ECT008	*CTNNB1, KMT2D/MLL4, PTEN*	9.8%	Histone modifier; PI(3)K signaling; WNT/β-catenin signaling
Good	ECT010	*CTNNB1, PIK3CA, PTEN, PTEN*	18.2%	PI(3)K signaling; WNT/β-catenin signaling
Good	ECT012	*CTNNB1, PTEN, PIK3CA*	18.1%	PI(3)K signaling; WNT/β-catenin signaling
Good	ECT013	*PIK3CA, PTEN, ATM, ARID1A*	11.7%	Histone modifier; Genome integrity; PI(3)K signaling
Good	ECT015	*CTNNB1, PTEN, PTEN, AR*	12.7%	PI(3)K signaling; WNT/β-catenin signaling; Other
Poor	EST007	*KRAS, TP53*	48.4%	Genome integrity; MAPK signaling
Poor	EST008	*PPP2R1A, KMT2D/MLL4, TP53, ARHGAP35*	21.4%	Histone modifier; Genome integrity; Protein phosphatase; Other
Poor	EST010	*PIK3CA, AKT1, TP53, ARHGAP35*	46.3%	Genome integrity; PI(3)K signaling; Other
Poor	EST011	*ARID1A, MAP3K1, SOX17, PTEN, TP53, PTEN, ARID1A, PIK3R1*	37.4%	Histone modifier; Genome integrity; MAPK signaling; PI(3)K signaling; WNT/β-catenin signaling
Poor	EST013	*PTEN, PIK3CA, PIK3CA, SOX17, ARID1A, FOXA2, ARID1A*	12.2%	Transcription factor/regulator; Histone modifier; PI(3)K signaling; WNT/β-catenin signaling
Poor	EST014	*SPOP, ERCC2*	37.5%	Genome integrity; Proteolysis
Poor	ECT007	*KRAS, AKT1, TP53*	2.8%	Genome integrity; MAPK signaling; PI(3)K signalling
B. Ovarian Carcinoma			
Sample		Mutational genes	MAF	Pathways involved
0ST002		*TP53, SF3B1, VHL, RAD21, ACVR1B*	15.9%	Transcription factor/regulator; Genome integrity; TGF- β signaling; Splicing
0ST003		*ATR, TP53, EPPK1, VEZF1, STAG2*	25.0%	Transcription factor/regulator; Genome integrity; Other
0ST004		*EPHA3, CRIPAK, TP53*	42.7%	Genome integrity; RTK signaling; Other
0ST005		*APC, TP53, NCOR1, CTCF, VEZF1, ARID1A*	19.9%	Transcription factor/regulator; Histone modifier; Genome integrity; WNT/β-catenin signaling; Other
0ST006		*TP53, RUNX1, EPPK1, ARID1A*	5.7%	Transcription factor/regulator; Histone modifier; Genome integrity; Other
0ST007		*TP53, BRCA1*	65.5%	Genome integrity
0ST009		*EGFR, EZH2, SMC3, TP53, EP300, TP53*	18.2%	Transcription factor/regulator; Histone modifier; Genome integrity; RTK signaling
0ST010		*PTEN, PTEN, TP53, PIK3R1, RAD21*	27.0%	Genome integrity; PI(3)K signaling
0ST011		*ATM, LRRK2, ARID1A, TP53, TP53, KEAP1, PRX, ARHGAP35, PDGFRA, EGFR, HGF, HGF, EPPK1, NOTCH1, NOTCH1, TAF1, RPL22*	3.3%	Transcription factor/regulator; Histone modifier; Genome integrity; RTK signaling; Proteolysis; Ribosome; Other
0ST012		*MALAT1, TP53*	28.8%	Genome integrity; Other
0ST013		*EPPK1*	3.0%	Other
0ST016		*RB1, TP53, CDK12, SMC3, LRRK2, NF1, SETD2*, *PDGFRA*	32.9%	Histone modifier; Genome integrity; RTK signaling; Cell cycle; MAPK signaling; Other
0ST017		*TP53*	31.5%	Genome integrity
0ST019		*PIK3CA, TP53, ARHGAP35, RPL22*	21.0%	Genome integrity; PI(3)K signaling; Ribosome; Other
0ST020		*TLR4, TP53*	12.6%	Genome integrity; PI(3)K signaling
0ST021		*HIST1H1C, TP53*	25.5%	Genome integrity; Histone
0ST022		*PIK3CA, TP53, PIK3CA, BRCA1, ARID1A, ARID1A*	20.3%	Histone modifier; Genome integrity; PI(3)K signaling

MAF, Mutant Alleles Fraction.

### Establishment of the EC Gene Panel and OC Gene Panel for Liquid Biopsy

According to the whole-exome sequencing data for the 127 driver genes in 22 EC/AHs and 17 OCs, 9 genes with 38 hot spot mutations in EC/AH and 8 genes with 29 hot spot mutations in OC were established as the gene panel for the cSMART test in Pap smear and plasma specimens. The gene panels of EC and OC are detailed in **Supplementary Material 1**.

### Spectrum of Mutations in Pap Smear DNA of EC/AH

**Figure 4:**
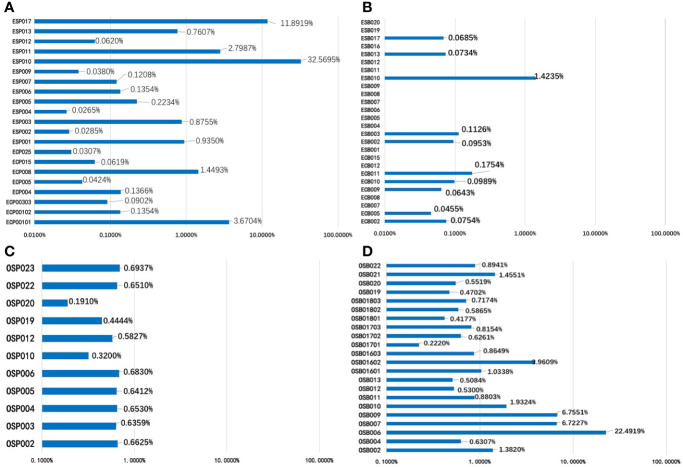
The mutant allele fractions (MAFs) of nine driver genes of the EC panel for 21 Pap smear specimens **(A)** and 26 plasma specimens **(B)**; the MAFs of eight driver genes of the OC panel in 11 Pap smear specimens **(C)** and 22 plasma specimens **(D)**.

**Figure 5:**
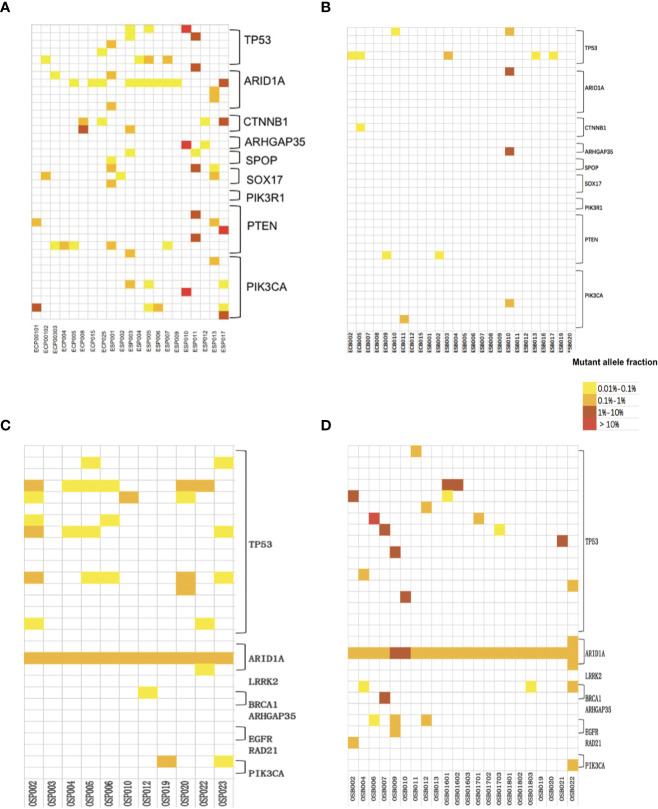
Heat map depicting the results of multiplex testing of nine driver genes in EC Pap smear specimens **(A)** and EC plasma specimens **(B)**; heat map depicting the results of multiplex testing of 8 driver genes in OC Pap smear specimens **(C)** and OC plasma specimens **(D)**. The cSMART test examines 38 gene regions in EC specimens or 29 gene regions in OC specimens, with each block on the y-axis representing one region analyzed for the indicated gene. The samples assessed are indicated on the x-axis. Mutations are indicated as colored blocks, with white indicating no mutation, yellow indicating a mutant fraction of 0.01 to 0.1%, orange indicating a mutant fraction of 0.1 to 1%, brown indicating a mutant fraction of 1–10%, and red indicating a mutant fraction of >10%.

In the EC/AH group, 21 Pap smear specimens, involving 20 patients, were included for cSMART multigene panel testing. The one more Pap smear specimen was repeatedly sampled in Patient 001. All Pap smear specimens tested positive (100%), with a median of 2 (1–7) mutated gene regions, 2 (1–6) genes and 0.1354% (0.0265–32.5695%) mutant allele fractions, as indicated in **Table 4** and **Figure 4A**. The heat map depicting the results of multiplex testing of the nine genes in the Pap smear specimens is presented in **Figure 5A**. Among the Pap smear specimens, 30 (78.9%, 30/38) mutated gene regions were detected in the 38 gene-region panel for EC.

**Table 4:** The mutated genes and mutant alleles fraction.

B. EC/AH-Plasma					
Patient	Tumor	Mutated genes in gene-panel	Plasma	Mutated genes	MAF
1	EST001 —	*ARID1A ARID1A PIK3R1* *PTEN SOX17 SOX17 SPOP TP53*	ESP001	*ARID1A ARID1A PTEN SOX17 SOX17 SPOP TP53*	0.9350%
2	EST002	none	ESP002	*SOX17*	0.0285%
3	EST003	*CTNNB1 PIK3CA PTEN*	ESP003	*ARID1A CTNNB1 PIK3CA PTEN SPOP TP53 TP53*	0.8755%
4	EST004	*CTNNB1 PIK3CA PTEN*	ESP004	*ARID1A TP53*	0.0265%
5	—	—	ESP005	*ARID1A PIK3CA PIK3CA TP53 TP53*	0.2234%
6	—	—	ESP006	*ARID1A PIK3CA*	0.1354%
7	EST007	*TP53*	ESP007	*ARID1A PTEN TP53*	0.1208%
8			ESP009	*ARID1A*	0.0380%
9	EST010	*ARHGAP35 PIK3CA TP53*	ESP010	*ARHGAP35 PIK3CA TP53*	32.5695%
10	EST011	*ARID1A PIK3R1 PTEN PTEN SOX17 TP53*	ESP011	*ARID1A PTEN PTEN SOX17 SPOP TP53*	2.7987%
11	—	—	ESP012	*ARHGAP35 CTNNB1*	0.0620%
12	EST013	*ARID1A ARID1A PIK3CA PIK3CA PTEN SOX17*	ESP013	*ARID1A ARID1A PIK3CA PTEN SOX17 SOX17*	0.7607%
13	EST017	*ARID1A CTNNB1 PIK3CA PTEN*	ESP017	*ARID1A CTNNB1 PIK3CA PIK3CA PIK3CA PTEN*	11.8919%
14	—	—	ECP00101	*PIK3CA PTEN*	3.6704%
			ECP00102	*SOX17 TP53*	0.1354%
15	—	—	ECP003	*ARID1A PTEN*	0.0902%
16	ECT004	*PIK3CA PTEN*	ECP004	*PTEN*	0.1366%
17	ECT005	*none*	ECP005	*ARID1A PTEN*	0.0424%
18	ECT008	*CTNNB1*	ECP008	*CTNNB1 CTNNB1*	1.4493%
19	ECT015	*none*	ECP015	*ARID1A*	0.0619%
20	—	—	ECP025	*ARID1A CTNNB1 TP53*	0.0307%
**B. EC/AH-Plasma**					
**Patient**	**Tumor**	**Mutated genes in gene-panel**	**Plasma**	**Mutated genes**	**MAF**
1	EST001	*ARID1A ARID1A PIK3R1* *PTEN SOX17 SOX17 SPOP TP53*	ESB001	*none*	none
2	EST002	*none*	ESB002	*PTEN*	0.0953%
3	EST003	*CTNNB1 PIK3CA PTEN*	ESB003	*TP53*	0.1126%
4	EST004	*CTNNB1 PIK3CA PTEN*	ESB004	*none*	none
5	—	—	ESB005	*none*	
6	—	—	ESB006	*none*	
7	EST007	*TP53*	ESB007	*none*	
8	EST008	*ARHGAP35 TP53*	ESB008	*none*	
9	—	—	ESB009	*none*	
10	EST010	*ARHGAP35 PIK3CA TP53*	ESB010	*ARHGAP35 ARID1A PIK3CA TP53*	1.4235%
11	EST011	*ARID1A PIK3R1 PTEN PTEN SOX17 TP53*	ESB011	*none*	
12	—	—	ESB012	*none*	
13	EST013	*ARID1A ARID1A PIK3CA PIK3CA PTEN SOX17*	ESB013	*TP53*	0.0734%
14	—	—	ESB016	*none*	
15	EST017	*ARID1A CTNNB1 PIK3CA PTEN*	ESB017	*TP53*	0.0685%
16	EST019	*none*	ESB019	*none*	
17	EST020	*PIK3CA PIK3CA PTEN PTEN*	ESB020	*none*	
18	ECT002	*PIK3CA*	ECB002	*TP53*	0.0754%
19	ECT005	*none*	ECB005	*CTNNB1 TP53*	0.0455%
20	ECT007	*none*	ECB007	*none*	
21	ECT008	*CTNNB1*	ECB008	*none*	
22	—	—	ECB009	*PTEN*	0.0643%
23	ECT010	*none*	ECB010	*TP53*	0.0989%
24	—	—	ECB011	*PIK3CA*	0.1754%
25	ECT012	*none*	ECB012	*none*	
26	ECT015	*none*	ECB015	*none*	
**C. OC-Pap smear**											
**Patient**	**Tumor**	**Mutated genes in gene-panel**	**Pap smear**	**Mutated genes**	**MAF**
1	OST002	*RAD21 TP53*	OSP002	*TP53 TP53 TP53 TP53 TP53 ARID1A ARID1A*	0.6625%
2	OST003	*TP53*	OSP003	*ARID1A*	0.6359%
3	OST004	*TP53*	OSP004	*TP53 TP53 ARID1A*	0.6530%
4	OST005	*TP53 ARID1A*	OSP005	*TP53 TP53 TP53 TP53 ARID1A*	0.6412%
5	OST006	*TP53 ARID1A*	OSP006	*TP53 TP53 TP53 ARID1A*	0.6830%
6	OST010	*TP53*	OSP010	*TP53 ARID1A*	0.3200%
7	OST012	*TP53*	OSP012	*BRCA1 ARID1A*	0.5827%
8	OST019	*TP53 PIK3CA ARHGAP35*	OSP019	*PIK3CA ARID1A*	0.4444%
9	OST020	*TP53*	OSP020	*TP53 TP53 TP53 TP53 ARID1A*	0.1910%
10	OST022	*TP53 PIK3CA BRCA1 ARID1A ARID1A*	OSP022	*TP53 TP53 ARID1A ARID1A*	0.6510%
11	—	—	OSP023	*TP53 TP53 TP53 ARID1A PIK3CA*	0.6937%
**D. OC-Plasma**											
**Patient**	**Tumor**	**Mutated genes in gene-panel**	**Plasma**	**Mutated genes**	**MAF**
1	OST002	*RAD21 TP53*	OSB002	*TP53 ARID1A RAD21*	1.3820%
2	OST004	*TP53*	OSB004	*TP53 ARID1A BRCA1*	0.6307%
3	OST006	*TP53 ARID1A*	OSB006	*TP53 ARID1A EGFR*	22.4919%
4	OST007	*TP53 BRCA1*	OSB007	*TP53 ARID1A BRCA1*	6.7227%
5	OST009	*TP53 TP53 EGFR*	OSB009	*TP53 ARID1A EGFR EGFR*	6.7551%
6	OST010	*TP53*	OSB010	*TP53 ARID1A*	1.9324%
7	OST011	*TP53 LRRK2 EGFR*	OSB011	*TP53 ARID1A*	0.8803%
8	OST012	*TP53*	OSB012	*TP53 ARID1A EGFR*	0.5300%
9	OST013	*none*	OSB013	*ARID1A*	0.5084%
10	OST016	*TP53 LRRK2*	OSB01601	*TP53 TP53 ARID1A*	1.0338%
			OSB01602	*TP53 ARID1A*	3.9609%
			OSB01603	*ARID1A*	0.8649%
11	OST017	*TP53*	OSB01701	*TP53 ARID1A*	0.2220%
			OSB01702	*ARID1A*	0.6261%
			OSB01703	*TP53 ARID1A*	0.8154%
12	—	—	OSB01801	*ARID1A*	0.4177%
			OSB01802	*ARID1A*	0.5865%
			OSB01803	*ARID1A BRCA1*	0.7174%
13	OST019	*TP53 PIK3CA ARHGAP35*	OSB019	*ARID1A*	0.4702%
14	OST020	*TP53*	OSB020	*ARID1A*	0.5519%
15	OST021	*TP53*	OSB021	*TP53 ARID1A*	1.4551%
16	OST022	*TP53 PIK3CA BRCA1 ARID1A ARID1A*	OSB022	*TP53 ARID1A ARID1A ARID1A PIK3CA BRCA1*	0.8941%

The mutated genes in liquid material concurrently with the tumor specimen were marked red.

Tumor tissue was available for 13 patients (13 Pap smear specimens) and not available for the other 7 patients (8 Pap smear specimens). Among the 13 tumors, 11 tumors contained hot spot mutations in the EC gene panel and 2 tumors not. Among the 11 corresponding Pap smear specimens, 9 (81.8%, 9/11) identified the same gene mutations with tumor tissues. The remaining 10 Pap smear specimens, which did not have matched tumor tissues (n = 8) or whose matched tumors did not contain hot spot mutations in the EC gene panel (n = 2), all tested positive. Additionally, 27 of 30 (90.0%) mutated gene regions detected in Pap smear specimens were identical to those observed in the matched primary tumor tissues.

### Spectrum of Mutations in Plasma ccfDNA of EC/AH

In the EC/AH group, 26 plasma specimens from 26 patients were included for cSMART multigene panel testing. Ten plasma specimens tested positive (10/26, 38.5%), with a median of 0 (0–4) mutated gene regions, 0 (0–4) genes and a 0.0854% (0.0455–1.4235%) mutant allele fraction, as detailed in **Table 4** and **Figure 4B**. The heat map depicting the results of multiplex testing of nine genes in plasma specimens is shown in **Figure 5B**. In plasma specimens, 8 (21.1%, 8/38) mutated gene regions were detected in the 38 gene-region panel of EC.

Tumor tissue was available from 19 patients and not available for the other 7 patients. Thirteen out of 19 tumors contained hot spot mutations in the EC gene panel and 6 tumors not. Plasma specimens identified gene mutations in 7.7% (1/13) of tumor tissues. Five (38.5%) out of the remaining 13 plasma specimens, which did not have matched tumor tissues (n = 7) or whose matched tumors did not contain hot spot mutations in the EC gene panel (n = 6), tested positive. Additionally, 3 out of 8 (37.5%) mutated gene regions detected in plasma specimens were identical to those observed in their primary tumor tissues.

### Spectrum of Mutations in Pap Smear DNA of OC

In the OC group, 11 Pap smear specimens, involving 11 patients, were included for cSMART multigene panel testing. All Pap smear specimens tested positive (100%), with a median of 4 (1–7) mutated gene regions, 2 (1–3) genes, and 0.6412% (0.1910–0.6937%) mutant allele fractions, as detailed in **Table 4** and **Figure 4C**. The heat map depicting the results of multiplex testing of eight genes in Pap smear specimens is depicted in **Figure 5C**. For the Pap smear specimens, 12 (41.4%, 12/29) mutated gene regions were detected in the 29 gene-region panel for OC.

Tumor tissue was available for 10 patients, and all 10 tumors contained hot spot mutations in the OC gene panel. Pap smear specimens identified gene mutations in 50.0% (5/10) of tumor tissues. The remaining Pap smear specimen, which did not have matched tumor tissue, also tested positive. Additionally, 6 of 12 (50.0%) mutated gene regions detected in Pap smear specimens were matched to those observed in their tumor tissues.

### Spectrum of Mutations in Plasma ccfDNA of OC

In the OC group, 22 plasma specimens, involving 16 patients (3 patients were sampled for three times), were included for cSMART multigene panel testing. All the plasma specimens tested positive (100%), with a median of 2 (1–6) mutated gene regions, 2 (1–4) genes, and 0.8402% (0.2220–22.4919%) mutant allele fraction, detailed in **Table 4** and **Figure 4D**. The heat map illustrating the results of multiplex testing of eight genes in plasma specimens is presented in **Figure 5D**. Of the plasma specimens, 20 (69.0%, 20/29) mutated gene regions were detected in the 29 gene-region panel of OC.

Tumor tissue was available for 15 patients. Fourteen of 15 tumors contained hot spot mutations in the OC gene panel. Plasma specimens identified gene mutations in 71.4% (10/14) of tumor tissues. The remaining four plasma specimens, which did not have matched tumor tissue (n = 3) or whose matched tumor did not contain hot spot mutations in the OC gene panel (n = 1), all tested positive. Additionally, 18 of 20 (90.0%) mutated gene regions detected in plasma specimens were identical to those observed in their primary tumor tissues.

**Figure 6:**
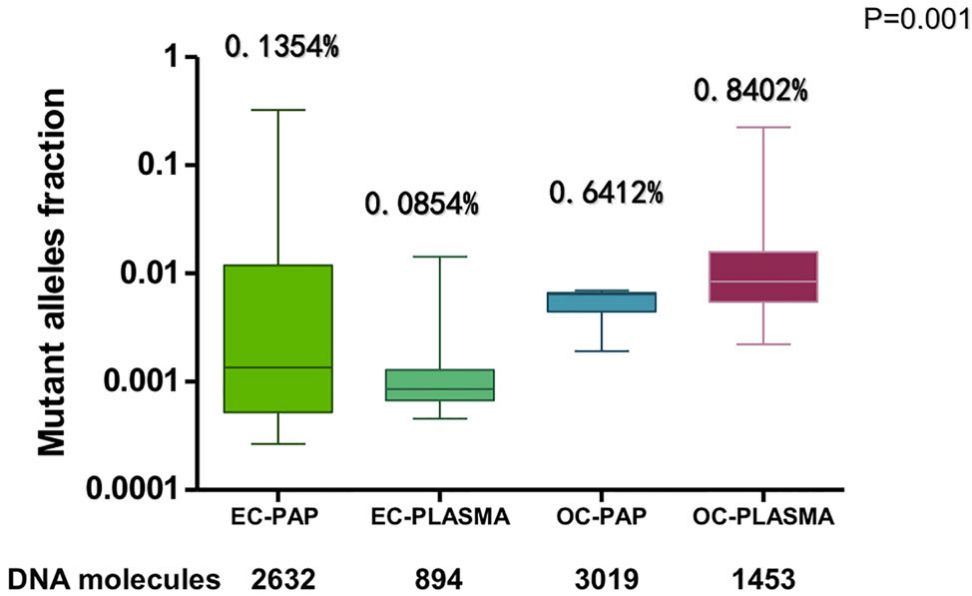
The MAFs of EC-Pap smear, EC-Plasma, OC-Pap smear, and OC-Plasma specimens.

The mutant allele fractions (MAFs) of Pap smear DNA and plasma ccfDNA in EC/AH or OC patients (EC-Pap smear, EC-plasma, OC-Pap smear, and OC-plasma) are presented in **Figure 6**. MAFs in the positive specimens of the EC-Pap smear, EC-plasma, OC-Pap smear, and OC-plasma were above 0.02, 0.04, 0.2, and 0.2%, respectively. The MAFs of EC-plasma were the lowest. Apparently, Pap smear was more useful for EC/AH than plasma, as EC-plasma exhibited low positivity and poor consistency. However, compared with the other three groups, the EC-Pap smear samples had the widest range of MAFs. In general, the MAFs of OC specimens were higher than those of EC/AH specimens, irrespective of the source, i.e., Pap smear or plasma. The OC-plasma specimens appeared to have relatively higher MAFs than the OC-Pap smear specimens.

## Discussion

Here, we present a small-sample, observational study of paired tumors and liquid-based materials of epithelial OC or EC/AH. Initially, patients in the gynecology clinic highly suspected of having an ovarian or endometrial malignancy were included in the study. To explore the role of liquid-based materials for liquid biopsy for EC and OC, we investigated Pap smears and peripheral blood because theoretically, neoplastic cells shed from an ovarian or endometrial malignancy can be collected at the cervix and all malignant tumors can release neoplastic cells into the circulation. All tumor tissues in our study were collected during surgery, and Pap smear specimens and peripheral blood specimens were collected before the operation. When gathering tumor mutation data, matched leukocyte data must be acquired simultaneously. Due to some uncontrollable factors, tumor and leukocyte pairs were acquired simultaneously for only 43 patients. After screening, 51 patients with pathologically confirmed epithelial OC or EC or AH, either with matched tumors and leukocytes, qualified plasma ccfDNA, or qualified Pap smear DNA, were included in the study.

TCGA data show that TP53 is the gene with the highest mutation frequency in all cancers, up to 42%, and accounts for the highest proportions of HGSOC, up to 94.5%, and serous EC, up to 89%. PIK3CA is the second most commonly mutated gene in all cancers and accounts for 52.2% of ECs (10). In our work, the incidence of TP53 gene mutation in epithelial OC was as high as 94.12%; it was highest in poorly differentiated EC at up to 71.43% and significantly higher than that in well-differentiated EC. The results for somatic mutations in EC or epithelial OC in our study were consistent with those reported by TCGA. Initially, we wondered whether atypical hyperplasia should be included in the study. It is important to differentiate between atypical hyperplasia and endometrial carcinoma. The relationship between somatic mutations and the grade of tumor malignancy is not known now and it is difficult to differentiate between atypical hyperplasia and endometrial carcinoma through different somatic mutations. However, somatic mutations occur at an early stage of cellular malignant transformation, especially precancerous stage. Endometrial atypical hyperplasia has been proved to be precancerous lesions of type I endometrial carcinoma. Therefore, to investigate the significant somatic mutations at a precancerous stage is of great importance for early detection of malignancy. That’s why atypical hyperplasia was included in the analysis.

All the CTNNB1 mutations in well-differentiated EC tumors led to accumulation of the gene product and activation of cell proliferation (reference to JAX-Clinical Knowledgebase annotation). In addition, the TP53 mutation and the CTNNB1 mutation were exclusive among tumors, which indicated that tumors with the TP53 mutation might have heterogeneous expansion clones and resemble the biological behavior of type II ECs. Tumors with CTNNB1 mutations might represent a lower stage of malignant proliferation, not yet entering the stage of infiltration and metastasis. Thus, we might reasonably deduce that the emergence of TP53 mutations predicts poorly differentiated EC tumors and that the emergence of CTNNB1 mutations predicts well-differentiated EC tumors.

In this study, mutant alleles were detected in all Pap smears from EC patients and had a good correlation with tumor tissues, with a coincidence rate of 81.8%. However, the detection rate of circulating tumor DNA (ctDNA) was poor among EC patients and had a poor correlation with tumor tissues, which may be related to the low tumor burden of early-stage EC. A large number of studies have confirmed that ctDNA correlates positively with tumor load (19–21). Mutant alleles were detected in all Pap smears and plasma specimens of OC patients, and also showed a good correlation with tumor tissues, with a coincidence rate of 50.0 and 71.4% in Pap smears and plasmas, respectively. The MAFs in OC increased by a logarithmic level compared with those in EC, which may be associated with the relatively higher tumor burden of OC than with EC.

From the perspective of the gene panel, the 127-driver gene panel from TCGA was able to detect significantly mutated genes in our small sample of EC and OC tumor tissues. Ultimately, 34 genes in EC and 41 genes in OC were mutated, and 9 genes in EC and 8 genes in OC were selected as the gene panel for the liquid material. Both the EC and OC gene panels covered the mutated gene regions of most tumor specimens. PIK3R1 was not detected in both EC-Pap smear and -plasma specimens, and *LRRK2* and *ARHGAP35* were not detected in OC-Pap smear and -plasma specimens; in addition, *SPOP* and *SOX17* were not detected in the EC-plasma specimens, and *EGFR* and *RAD21* were not detected in the OC-Pap smears. It is necessary to establish a unified and consistent Pap smear gene panel for EC and OC. Indeed, tumor cells of both EC and OC can be shed in the cervix and collected. For plasma ccfDNA, a variety of tumors can release tumor cells into the circulation; thus, creating different gene panels is unlikely to distinguish different malignancies. Gene panels are more widely used for the early detection of malignancies than for distinguishing a specific type of tumor. Pap smears are a perfect tool for liquid biopsy for EC, whereas collecting both Pap smear and plasma specimens might be more efficient for OC.

Tumor heterogeneity and the limitations of tumor tissue sampling led to the difference in somatic mutations between ctDNA and tumor tissue; that is, the ctDNA assays identified not only mutations known to be present in tumors but also a large number of variants absent in the respective tumor tissues (22). Researchers have found that ctDNA has good consistency with the gene mutations detected in tumor tissues, despite the influence of tissue sampling limitations and other factors (23). ctDNA sequencing can accurately detect a large number of mutations in the tumor genome, reflecting the tumor mutational burden and tumor mutational signatures, and can detect subclonal mutations that cannot be detected by tumor biopsy (22). Due to the limited coverage of nine-gene EC or -gene OC panel, the definite tumor-derived DNA was not found in the remaining 50% Pap smears and 29% ccfDNA from OC patients and 18% Pap smears from EC/AH patients. It’s expected that at least one same mutation would be detected in each Pap smear or ccfDNA as that from tumor DNA, if we expanded the number of genes or loci for testing or established a specific testing panel according to the matched tumor itself. In our study, gene mutations in one patient with mild atypical hyperplasia were not detected in the patient’s tumor tissue, but gene mutations in Pap smear DNA and plasma ccfDNA were detected.

The following limitations that need to be addressed. First, in terms of specimen collection, sufficient DNA cannot be extracted from many Pap smear specimens to meet the requirements of assays. The procedure and execution of blood sampling need to be optimized and further standardized to avoid hemolysis. Second, the effect of clonal hematopoiesis (CH) on accurately identifying tumor-derived DNA cannot be completely avoided. There are a number of ctDNA variations that are consistent with CH. In our study, we performed whole-exome sequencing of 127 genes in both tumor tissues and matched leukocytes, theoretically filtering out a large number of clonal hematopoietic variants. However, to efficiently avoid the effect of CH, matched leukocyte sequencing should be performed at similar depths to those employed for ctDNA analysis. Regarding Pap smear DNA, a gold standard of identifying tumor cells in these specimens may be established if the cell pellets collected through the Pap smear are diagnosed under the microscope by experienced pathologists or cytologists before processing to obtain DNA. In this study, OC cells from the Pap smear specimens of three OC patients were identified by our experienced cytologist. Finally, a large-scale prospective cohort study among normal populations or patients with benign tumors or cervical lesions or malignant Mullerian duct tumors in a blind state is required to evaluate the sensitivity and specificity of the testing method to guide the early diagnosis and detection of malignant Mullerian duct tumors. Next, we try to establish a more accurate, reliable, and targeted gene panel, which can cover the female genital tract malignant tumors. Therefore, the evaluation of gene mutations in the female genital tract malignant tumors should also be carried out. The establishment of a more accurate gene panel will lay the foundation for the establishment of its universality in the next step.

## Conclusions

Using the 127-driver gene panel of TCGA and whole-exome sequencing, significantly mutated genes in EC and OC tumor tissues were confirmed, and eight- and nine-gene fluid testing panels for EC and OC, respectively, were established. Based on cSMART, somatic mutations carrying tumor genome information were detected in 100% of EC-Pap smear DNA, 100% of OC-Pap smear DNA, and 100% of OC-plasma ccfDNA. Pap smear DNA and plasma ccfDNA might be expected to provide feasible material for noninvasive liquid biopsy for the monitoring of tumor status in patients with EC or OC and may even provide clues for the early detection of EC and OC. However, the result with plasma ccfDNA in EC is poor, and Pap smear DNA may be the only effective noninvasive liquid biopsy method to monitor EC.

## Data Availability Statement

The original contributions presented in the study are included in the article/**Supplementary Material**. Further inquiries can be directed to the corresponding authors.

## Ethics Statement

The studies involving human participants were reviewed and approved by Ethics Committee of Peking Union Medical College Hospital. The patients/participants provided their written informed consent to participate in this study.

## Author Contributions

XJ: conception, design, data interpretation, drafting, revisions, final approval. WL: design, revisions, final approval. JY: design, data acquisition, revisions, final approval. SW: data acquisition, revisions, final approval. DC: conception, data interpretation, revisions, final approval. MY: data analysis and interpretation, drafting, revisions, final approval. KS: conception, design, data interpretation, revisions, final approval. JB: data interpretation, revisions, final approval. YG: conception, design, data acquisition and interpretation, revisions, final approval. All authors contributed to the article and approved the submitted version.

## Funding

This study was supported by the Chinese Academy of Medical Sciences Initiative for Innovative Medicine (CAMS-2017-I2M-1-002), the Beijing Science and Technology Ministry of Capital Citizens’ Health Project Cultivation (Z161100000116077), National Natural Science Foundation of China (81702551), and Beijing Postdoctoral Research Foundation (2018-ZZ-106).

## Conflict of Interest

Author JB and author YG were employed by the company Berry Oncology.

The remaining authors declare that the research was conducted in the absence of any commercial or financial relationships that could be construed as a potential conflict of interest.
