# Supplementary material for: Identification of Somatic Mutations in Papanicolaou Smear DNA and Plasma Circulating Cell-Free DNA for Detection of Endometrial and Epithelial Ovarian Cancers: A Pilot Study
Source: Front Oncol. 2020 Dec 14;10:582546. doi: 10.3389/fonc.2020.582546 (PMC7768029; doi:10.3389/fonc.2020.582546)
Supplement: Supplementary file 1 [file DataSheet_1.docx]

**Supplementary material-1**

**Contents list:**

1. **Table 1. 127-gene list and their mutational frequency in ovarian and endometrial cancers in TCGA and our study**
2. **Table 2. The primer sequence of EC and OC gene-panel in cSMART technology**
3. **Table 3. EC and OC gene-panel for liquid biopsy in cSMART technology**
4. **Table 4. The quality control of the sequencing data of tumor tissues and leukocytes**
5. **Table 5. The TP53 mutations and CTNNB1 mutations in EC tumors**

**Table 1. 127-gene list and their mutational frequency in ovarian and endometrial cancers**

**in TCGA and our study**

| **Gene** | **OC Frequency-TCGA** | **EC Frequency-TCGA** | **OC Frequency-Our study** | **EC Frequency- Our study** |
| --- | --- | --- | --- | --- |
| **PTEN** | 0.60% | 63.50% | 5.88% | 59.09% |
| **PIK3CA** | 0.60% | 52.20% | 11.76% | 50.00% |
| **PIK3R1** | 0.30% | 30.90% | 5.88% | 9.09% |
| **ARID1A** | 1.00% | 30.00% | 23.53% | 27.27% |
| **CTNNB1** | 0.60% | 28.30% | 0.00% | 36.36% |
| **TP53** | 94.60% | 27.80% | 94.12% | 27.27% |
| **KRAS** | 0.60% | 20.00% | 0.00% | 9.09% |
| **CTCF** | 0.30% | 16.50% | 5.88% | 4.55% |
| **FBXW7** | 1.00% | 11.70% | 0.00% | 0.00% |
| **RPL22** | 0.00% | 10.90% | 11.76% | 0.00% |
| **FGFR2** | 0.00% | 10.40% | 0.00% | 4.55% |
| **ARHGAP35** | 1.60% | 10.00% | 11.76% | 9.09% |
| **ARID5B** | 0.60% | 9.60% | 0.00% | 4.55% |
| **TAF1** | 1.60% | 8.70% | 5.88% | 9.09% |
| **PPP2R1A** | 1.30% | 8.70% | 0.00% | 4.55% |
| **MLL2** | 0.60% | 8.30% | 0.00% | 9.09% |
| **MLL4** | 0.30% | 8.30% | 0.00% | 4.55% |
| **ATR** | 0.60% | 7.00% | 5.88% | 0.00% |
| **ATM** | 1.30% | 6.50% | 5.88% | 4.55% |
| **SPOP** | 0.30% | 6.50% | 0.00% | 9.09% |
| **USP9X** | 0.30% | 6.50% | 0.00% | 0.00% |
| **APC** | 2.20% | 5.70% | 5.88% | 0.00% |
| **NSD1** | 0.60% | 5.70% | 0.00% | 0.00% |
| **MLL3** | 1.90% | 5.20% | 0.00% | 9.09% |
| **MTOR** | 1.90% | 5.20% | 0.00% | 0.00% |
| **NAV3** | 1.30% | 5.20% | 0.00% | 4.55% |
| **SIN3A** | 0.60% | 5.20% | 0.00% | 4.55% |
| **EP300** | 0.30% | 5.20% | 5.88% | 4.55% |
| **CCND1** | 0.00% | 5.20% | 0.00% | 0.00% |
| **NFE2L2** | 0.00% | 5.20% | 0.00% | 0.00% |
| **FOXA2** | 0.60% | 4.80% | 0.00% | 4.55% |
| **BRCA2** | 3.20% | 4.40% | 0.00% | 0.00% |
| **SMC1A** | 1.30% | 4.40% | 0.00% | 0.00% |
| **RB1** | 1.90% | 3.90% | 5.88% | 0.00% |
| **POLQ** | 1.00% | 3.90% | 0.00% | 0.00% |
| **STAG2** | 1.00% | 3.90% | 5.88% | 0.00% |
| **TSHZ3** | 1.00% | 3.90% | 0.00% | 0.00% |
| **NF1** | 3.80% | 3.50% | 5.88% | 0.00% |
| **LRRK2** | 2.90% | 3.50% | 11.76% | 0.00% |
| **AR** | 0.30% | 3.50% | 0.00% | 9.09% |
| **MAP3K1** | 0.30% | 3.50% | 0.00% | 9.09% |
| **ATRX** | 0.60% | 3.00% | 0.00% | 0.00% |
| **MECOM** | 0.60% | 3.00% | 0.00% | 0.00% |
| **CDH1** | 0.30% | 3.00% | 0.00% | 0.00% |
| **EPPK1** | 0.30% | 3.00% | 23.53% | 9.09% |
| **SOX17** | 0.00% | 3.00% | 0.00% | 13.64% |
| **SETD2** | 1.90% | 2.60% | 5.88% | 0.00% |
| **NRAS** | 0.60% | 2.60% | 0.00% | 0.00% |
| **AXIN2** | 0.30% | 2.60% | 0.00% | 0.00% |
| **HIST1H2BD** | 0.30% | 2.60% | 0.00% | 0.00% |
| **PBRM1** | 0.30% | 2.60% | 0.00% | 0.00% |
| **ERBB4** | 0.00% | 2.60% | 0.00% | 0.00% |
| **CDK12** | 2.90% | 2.20% | 5.88% | 0.00% |
| **KDM5C** | 1.90% | 2.20% | 0.00% | 0.00% |
| **KIT** | 1.90% | 2.20% | 0.00% | 4.55% |
| **EPHA3** | 1.00% | 2.20% | 5.88% | 4.55% |
| **BAP1** | 0.60% | 2.20% | 0.00% | 0.00% |
| **SETBP1** | 0.00% | 2.20% | 0.00% | 0.00% |
| **SF3B1** | 0.00% | 2.20% | 5.88% | 0.00% |
| **TET2** | 0.00% | 2.20% | 0.00% | 0.00% |
| **TSHZ2** | 1.00% | 1.70% | 0.00% | 0.00% |
| **LIFR** | 0.60% | 1.70% | 0.00% | 4.55% |
| **NOTCH1** | 0.60% | 1.70% | 5.88% | 9.09% |
| **ACVR1B** | 0.30% | 1.70% | 5.88% | 0.00% |
| **EPHB6** | 0.30% | 1.70% | 0.00% | 0.00% |
| **NFE2L3** | 0.30% | 1.70% | 0.00% | 0.00% |
| **EZH2** | 0.00% | 1.70% | 5.88% | 0.00% |
| **EGFR** | 1.90% | 1.30% | 11.76% | 0.00% |
| **DNMT3A** | 1.00% | 1.30% | 0.00% | 0.00% |
| **PDGFRA** | 1.00% | 1.30% | 11.76% | 0.00% |
| **PIK3CG** | 1.00% | 1.30% | 0.00% | 0.00% |
| **TBX3** | 1.00% | 1.30% | 0.00% | 0.00% |
| **TGFBR2** | 1.00% | 1.30% | 0.00% | 0.00% |
| **EIF4A2** | 0.60% | 1.30% | 0.00% | 0.00% |
| **HGF** | 0.60% | 1.30% | 5.88% | 0.00% |
| **CHEK2** | 0.30% | 1.30% | 0.00% | 0.00% |
| **KEAP1** | 0.30% | 1.30% | 5.88% | 0.00% |
| **MAP2K4** | 0.30% | 1.30% | 0.00% | 0.00% |
| **NCOR1** | 0.30% | 1.30% | 5.88% | 0.00% |
| **PHF6** | 0.30% | 1.30% | 0.00% | 0.00% |
| **PRX** | 0.30% | 1.30% | 5.88% | 0.00% |
| **TBL1XR1** | 0.30% | 1.30% | 0.00% | 0.00% |
| **AKT1** | 0.00% | 1.30% | 0.00% | 9.09% |
| **RUNX1** | 0.00% | 1.30% | 5.88% | 0.00% |
| **SMAD2** | 0.00% | 1.30% | 0.00% | 0.00% |
| **BRCA1** | 3.50% | 0.90% | 11.76% | 0.00% |
| **FLT3** | 1.00% | 0.90% | 0.00% | 0.00% |
| **BRAF** | 0.60% | 0.90% | 0.00% | 0.00% |
| **CDKN1B** | 0.30% | 0.90% | 0.00% | 0.00% |
| **PTPN11** | 0.30% | 0.90% | 0.00% | 0.00% |
| **RAD21** | 0.30% | 0.90% | 11.76% | 0.00% |
| **ASXL1** | 0.00% | 0.90% | 0.00% | 0.00% |
| **B4GALT3** | 0.00% | 0.90% | 0.00% | 0.00% |
| **H3F3C** | 0.00% | 0.90% | 0.00% | 0.00% |
| **IDH1** | 0.00% | 0.90% | 0.00% | 0.00% |
| **KDM6A** | 0.00% | 0.90% | 0.00% | 0.00% |
| **PCBP1** | 0.00% | 0.90% | 0.00% | 0.00% |
| **RPL5** | 0.00% | 0.90% | 0.00% | 0.00% |
| **U2AF1** | 0.00% | 0.90% | 0.00% | 0.00% |
| **VHL** | 0.00% | 0.90% | 5.88% | 0.00% |
| **TLR4** | 1.00% | 0.40% | 5.88% | 0.00% |
| **ELF3** | 0.30% | 0.40% | 0.00% | 0.00% |
| **ERCC2** | 0.30% | 0.40% | 0.00% | 9.09% |
| **FGFR3** | 0.30% | 0.40% | 0.00% | 0.00% |
| **GATA3** | 0.30% | 0.40% | 0.00% | 4.55% |
| **MAPK8IP1** | 0.30% | 0.40% | 0.00% | 0.00% |
| **SMC3** | 0.30% | 0.40% | 11.76% | 0.00% |
| **ACVR2A** | 0.00% | 0.40% | 0.00% | 0.00% |
| **CBFB** | 0.00% | 0.40% | 0.00% | 0.00% |
| **CDKN2A** | 0.00% | 0.40% | 0.00% | 0.00% |
| **CRIPAK** | 0.00% | 0.40% | 5.88% | 0.00% |
| **EGR3** | 0.00% | 0.40% | 0.00% | 0.00% |
| **IDH2** | 0.00% | 0.40% | 0.00% | 0.00% |
| **NPM1** | 0.00% | 0.40% | 0.00% | 0.00% |
| **SOX9** | 0.00% | 0.40% | 0.00% | 0.00% |
| **STK11** | 0.00% | 0.40% | 0.00% | 0.00% |
| **WT1** | 0.00% | 0.40% | 0.00% | 0.00% |
| **HIST1H1C** | 1.30% | 0.00% | 5.88% | 0.00% |
| **MALAT1** | 1.00% | 0.00% | 5.88% | 9.09% |
| **CDKN2C** | 0.60% | 0.00% | 0.00% | 0.00% |
| **CDKN1A** | 0.30% | 0.00% | 0.00% | 0.00% |
| **AJUBA** | 0.00% | 0.00% | 0.00% | 0.00% |
| **CEBPA** | 0.00% | 0.00% | 0.00% | 0.00% |
| **FOXA1** | 0.00% | 0.00% | 0.00% | 0.00% |
| **MIR142** | 0.00% | 0.00% | 0.00% | 0.00% |
| **SMAD4** | 0.00% | 0.00% | 0.00% | 0.00% |
| **VEZF1** | 0.00% | 0.00% | 11.76% | 0.00% |

**Table 2. The Primer Sequence of EC and OC gene-panel in cSMART technology**

| **EC** | | |
| --- | --- | --- |
| **Primer No.** | **Primer Name** | **Primer Sequence(5'-3')** |
| 1 | ESPTENE7P1F | TTCCTCCAATTCAGGACCC |
|  | ESPTENE7P1R | CTTCACCTTTAGCTGGCAGAC |
|  | ESPTENE7P2F | ATCACCACACACAGGTAACGG |
|  | ESPTENE7P2R | TGGTGATATCAAAGTAGAGTTCTTCC |
| 2 | ESPTENE6P1F | CCAGTCAGAGGCGCTATGTG |
|  | ESPTENE6P1R | GGGAATAGTTACTCCctggtgg |
|  | ESPTENE6P2F | GCCACTGGTCTATAATCCAGATG |
|  | ESPTENE6P2R | GGCACTGTTGTTTCACAAGATG |
| 3 | ESPTENE5P1F | AAGATGACAATCATGTTGCAGC |
|  | ESPTENE5P1R | TCACTTAGCCATTGGTCAAGATC |
|  | ESPTENE5P2F | GGGGCAAATTTTTAAAGGCA |
|  | ESPTENE5P2R | GCACAAGAGGCCCTAGATTTC |
| 4 | ESPTENE3P1F | TTTAAGGTTTTTGGATTCAAAGC |
|  | ESPTENE3P1R | CAAACAAAAAGCCACCATTAAC |
|  | ESPTENE3P2F | TGTTTTAGAAGATATTTGCAAGCATAC |
|  | ESPTENE3P2R | CAACTATTAAGTGAAAGTTATCTGCTTG |
| 5 | ESPTENE1P1F | CATCATCAAAGAGATCGTTAGCAG |
|  | ESPTENE1P1R | ATGGCTGTCATGTCTGGGAG |
|  | ESPTENE1P2F | TCAAGTCTAAGTCGAATCCATCC |
|  | ESPTENE1P2R | ACCTGTATCCATTTCTGCGG |
| 6 | ESPIK3CAE2P1F | CCTTCGGCTTTTTCAACCC |
|  | ESPIK3CAE2P1R | AGGTCACAAAGTCGTCTTGTTTC |
|  | ESPIK3CAE2P2F | ATTGAGGATCTTTTCTTCACGG |
|  | ESPIK3CAE2P2R | CGAGAAATTGGTATGATACAATATCC |
| 7 | ESPIK3CAE10P1F | GAGATCCTCTCTCTGAAATCACTG |
|  | ESPIK3CAE10P1R | CGTGTAGAAATTGCTTTGAGCTG |
|  | ESPIK3CAE10P2F | ACTTACCTGTGACTCCATAGAAAATC |
|  | ESPIK3CAE10P2R | TAAGTGCTAAAATGGAGATTCTCTG |
| 8 | ESPIK3CAE12P1F | CTTGGTAAAAGATTGGCCTCC |
|  | ESPIK3CAE12P1R | CAAGCAATACATCTGTGTATGAGAAAG |
|  | ESPIK3CAE12P2F | CCAGAAGTTCCATAGCCTGTTC |
|  | ESPIK3CAE12P2R | GACTGTAATTACCCAGATCCTATGG |
| 9 | ESPIK3CAE19P1F | CCATTGACCTGTTTACACGTTC |
|  | ESPIK3CAE19P1R | AATGGCTGCATCATATCTACAAAG |
|  | ESPIK3CAE19P2F | AATGAAGGTAGCTACACAGTATCCAG |
|  | ESPIK3CAE19P2R | GGAATTGGAGATCGTCACAATAG |
| 10 | ESPIK3CAE20P1F | CAATCTTTTGATGACATTGCATAC |
|  | ESPIK3CAE20P1R | AGTTCTGGCATTCCAGAGCC |
|  | ESPIK3CAE20P2F | AGATCCAATCCATTTTTGTTGTC |
|  | ESPIK3CAE20P2R | TCCACACAATTAAACAGCATGC |
| 11 | ESARID1AE1P1F | GACTACAGTGGCGGGCC |
|  | ESARID1AE1P1R | CGGGGTAGCCCTGGTAG |
| 12 | ESARID1AE16P1F | GTATTCTCCTAGCCGCTACCC |
|  | ESARID1AE16P1R | AGTCTGGGTTGGAAGGCATG |
|  | ESARID1AE16P2F | ACCAGGCTTTACTCACCGTTG |
|  | ESARID1AE16P2R | TCTCGGTGCTGCTATGGATC |
| 13 | ESARID1AE18P1F | ACTGCCACAGCTGCTACTGAG |
|  | ESARID1AE18P1R | GCAGGATAGGCATTGCCATAC |
|  | ESARID1AE18P2F | CTGGGCATTGGTGCCAG |
|  | ESARID1AE18P2R | AACATGCCACCACAAATGATG |
| 14 | ESARID1AE20P1F | ATATCCAGACCCACTTCGAGAG |
|  | ESARID1AE20P1R | CTGGATATGCTCAGTGGTGTCC |
|  | ESARID1AE20P2F | TGTTGTCACATGCTTCCGAG |
|  | ESARID1AE20P2R | AGGGTACACCAGGGACAACAG |
| 15 | ESARID1AE20P3F | CTTCCTAGAGGACAGCCTTGC |
|  | ESARID1AE20P3R | CAGGAGGTTGCCGATACTG |
|  | ESARID1AE20P4F | TTCTGCATGTGGAGGAGGC |
|  | ESARID1AE20P4R | ACCCTTTGAGCCAACTAGTGTG |
| 16 | ESARID1AE20P5F | CGTTGATGAACTCATTGGTTTC |
|  | ESARID1AE20P5R | GTGATACCGAGATGTCCAACAG |
|  | ESARID1AE20P6F | GCTGTCATGACTGGCCAATC |
|  | ESARID1AE20P6R | ATGACAGCCGTGGGACACCTCC |
| 17 | ESTP53E6P1F | TTAGGTCTGGCCCCTCCTC |
|  | ESTP53E6P1R | GAGCAATCAGTGAGGAATCAGAG |
|  | ESTP53E6P2F | AGACCTCAGGCGGCTCATAG |
|  | ESTP53E6P2R | CTGAGGTCTGGTTTGCAACTG |
| 18 | ESTP53E7P1F | CCTGTGTTATCTCCTAGGTTGGC |
|  | ESTP53E7P1R | AAGATGAGGCCAGTGCGC |
|  | ESTP53E7P2F | TGATGATGGTGAGGATGGG |
|  | ESTP53E7P2R | TCATCACACTGGAAGACTCCAG |
| 19 | ESTP53E8P1F | AATCTACTGGGACGGAACAGC |
|  | ESTP53E8P1R | ACCACTACTCAGGATAGGAAAAGAG |
|  | ESTP53E8P2F | TCTCTCCCAGGACAGGCAC |
|  | ESTP53E8P2R | CACAGAGGAAGAGAATCTCCG |
| 20 | ESSOX17E1P1F | AGCAGAATCCAGACCTGCAC |
|  | ESSOX17E1P1R | GCTTGCGCTCGTCCTTAG |
|  | ESSOX17E1P2F | CACCCAGCATCTTGCTCAAC |
|  | ESSOX17E1P2R | GTGAGTCCGAGTCGCAGAC |
| 21 | ESSOX17E2P1F | CAGGACCACCCCAACTACAAG |
|  | ESSOX17E2P1R | GTCCTGCATGTGCTGCAC |
| 22 | ESSOX17E2P3F | AGGTCTCGGACTACGCTGG |
|  | ESSOX17E2P3R | TGCGCGTAGCTGTAGGTG |
| 23 | ESCTNNB1E1P1F | TCACTGGCAGCAACAGTCTTAC |
|  | ESCTNNB1E1P1R | TAACAGCCGCTTTTCTGTCTG |
|  | ESCTNNB1E1P2F | AAGGAGCTGTGGTAGTGGCAC |
|  | ESCTNNB1E1P2R | TGAGTGGTAAAGGCAATCCTG |
| 24 | ESPIK3R1E11P1F | TTACATGAATATAACACTCAGTTTCAAG |
|  | ESPIK3R1E11P1R | CTACAGCTTCAATATTATCTTCTTTGAC |
| 25 | ESSPOPE6P1F | GCTGTCCAAAGAGTGAAGTTCG |
|  | ESSPOPE6P1R | AGCTGACCAGTAACAGGTAAAGTG |
|  | ESSPOPE6P2F | TGAACAAAGAGGAGAACATTTACC |
|  | ESSPOPE6P2R | CAACTCTTAAGTTTCACATCCAGAAG |
| 26 | ESARHGAP35E1P1F | CATGGATATTTATGGCAAACACC |
|  | ESARHGAP35E1P1R | TATACAGATTCCTCCAGCCACTG |
|  | ESARHGAP35E1P2F | CTTAGCATCCAGCTCCAGTTC |
|  | ESARHGAP35E1P2R | CCCAGCAAGGAGAAGATGG |
| 27 | ESARHGAP35E1P3F | CAGCCAAACGTAAGGCCTC |
|  | ESARHGAP35E1P3R | ACAATGTACCCATGAACCAACC |
|  | ESARHGAP35E1P4F | CCTGCACTTCACAAAGAAAGG |
|  | ESARHGAP35E1P4R | TCCCTATTCAGCTTGTAGCACTC |

| **OC** | | |
| --- | --- | --- |
| **Primer No.** | **Primer Name** | **Primer Sequence(5'-3')** |
| 1 | OSTP53E06P01F | CTCCTAGGTTGGCTCTGACTG |
|  | OSTP53E06P01R | gagataacacaggcccaagatg |
|  | OSTP53E06P02F | GTGTGATGATGGTGAGGATGG |
|  | OSTP53E06P02R | TCCAGGTCAGGAGCCACTTG |
| 2 | OSTP53E07P01F | GGGACGGAACAGCTTTGAG |
|  | OSTP53E07P01R | CCCAGTAGATTACCACTACTCAGG |
| 3 | OSTP53E08P02F | CTTCTTTGGCTGGGGAGAG |
|  | OSTP53E08P02R | CACTGGATGGAGAATATTTCACC |
| 4 | OSTP53E03P01F | TCCCCGGACGATATTGAAC |
|  | OSTP53E03P01R | GGGACAGCATCAAATCATCC |
|  | OSTP53E03P02F | TCATCTGGACCTGGGTCTTC |
|  | OSTP53E03P02R | CTCCCAGAATGCCAGAGG |
| 5 | OSTP53E03P03F | CTACCAGGGCAGCTACGG |
|  | OSTP53E03P03R | CTGGGAAGGGACAGAAGATG |
|  | OSTP53E03P04F | GTCCCAGAATGCAAGAAGCC |
|  | OSTP53E03P04R | GCCAAGTCTGTGACTTGCAC |
| 6 | OSTP53E04P01F | TCCCCTGCCCTCAACAAG |
|  | OSTP53E04P01R | CTGTAGGAAGAGGAAGGAGACAG |
|  | OSTP53E04P02F | CTGCTTGTAGATGGCCATGG |
|  | OSTP53E04P02R | TCACAGCACATGACGGAGG |
| 7 | OSTP53E04P03F | GAATCAGAGGCCTGGGGAC |
|  | OSTP53E04P03R | CACTGATTGCTCTTAGGTCTGG |
| 8 | OSTP53E05P02F | GTTGCAAACCAGACCTCAGG |
|  | OSTP53E05P02R | GGGGTCTCTGGGAGGAGG |
| 9 | OSARID1AE11P01F | GTGTCTGTGAAGGAGATTGGTG |
|  | OSARID1AE11P01R | GCGATAGAGGTCCAGAGGTTTC |
|  | OSARID1AE11P02F | gatgatgtccctgtgggatg |
|  | OSARID1AE11P02R | CCTCTCCCGCTTTCTGAGTC |
| 10 | OSARID1AE16P01F | GATGTATTCTCCTAGCCGCTACC |
|  | OSARID1AE16P01R | GTCTGGGTTGGAAGGCATG |
|  | OSARID1AE16P02F | CGAGACCAGGCTTTACTCACC |
|  | OSARID1AE16P02R | CTGCTATGGATCAGGCTTCG |
| 11 | OSARID1AE20P01F | CCATCAAGGAGAGCAGCAAG |
|  | OSARID1AE20P01R | CTGAACTCTTAGCTCCATCCTCG |
|  | OSARID1AE20P02F | CAAGAGAATCCTGCCAGTCCAG |
|  | OSARID1AE20P02R | AGCGCTGCGTCTGTGTGTC |
| 12 | OSPIK3CAE09P01F | CGAGATCCTCTCTCTGAAATCAC |
|  | OSPIK3CAE09P01R | CTCGTGTAGAAATTGCTTTGAGC |
|  | OSPIK3CAE09P02F | CTTACCTGTGACTCCATAGAAAATC |
|  | OSPIK3CAE09P02R | GCTAAAATGGAGATTCTCTGTTTC |
| 13 | OSBRCA1E01P01F | GTTCATTGGAACAGAAAGAAATGG |
|  | OSBRCA1E01P01R | CCAATGAACTTTAACACATTAGAAAAAC |
|  | OSBRCA1E01P02F | GACATTTTGTACTTCTTCAACGCG |
|  | OSBRCA1E01P02R | GCTATGCAGAAAATCTTAGAGTGTC |
| 14 | OSBRCA1E16P01F | GTTTGTGTGTGAACGGACACTG |
|  | OSBRCA1E16P01R | CAAACTCAGCATctgcagaatg |
|  | OSBRCA1E16P02F | ggtgtaaaaatgcaattctgagg |
|  | OSBRCA1E16P02R | TCACCTAACGTTTAACACCTAAGG |
| 15 | OSEGFRE05P01F | GACCAAAATCATCTGTGCCCAG |
|  | OSEGFRE05P01R | GTCActgaaaaagagcaaaggttc |
|  | OSEGFRE05P02F | tcttacCAGGCAGTCGCTCTC |
|  | OSEGFRE05P02R | ATGCCCCTCCAGCAGCCTC |
| 16 | OSEGFRI17P01F | CAAAAAGATCAAAGTGCTGGG |
|  | OSEGFRI17P01R | ATTCAGTTTCCTTCAAGATCCTC |
|  | OSEGFRI17P02F | aggactctgggctccccac |
|  | OSEGFRI17P02R | GCAAGCTGTATATTTCCATCATC |
| 17 | OSARHGAP35E1P01F | GCTCAAGTTTGTCTCCAATCTCTAC |
|  | OSARHGAP35E1P01R | CATCAAAGTTCCTATTCATGCCC |
|  | OSARHGAP35E1P02F | CGCTCAACACCTTCGTCAC |
|  | OSARHGAP35E1P02R | GCGGTACATTAGAGATGCACATAC |
| 18 | OSLRRK2E32P01F | GAGAAAATCAGCTGCAGTTAGATG |
|  | OSLRRK2E32P01R | GTTGTAATAATCGTTTCCGGTC |
| 19 | OSRAD21E01P01F | ATAGCCAGCCAGAACAATGTTC |
|  | OSRAD21E01P01R | GGCTATGAAAACAGAAGAAAACc |
|  | OSRAD21E01P02F | GGTTAGCTTCTTATCCCAATGGG |
|  | OSRAD21E01P02R | AAGCCCATGTGTTCGAGTG |

**Table 3-A. EC gene-panel**

| **Gene** | **Transcript ID** | **Nucleotide (genomic)** | **Exon#** | **cDNA Change** | **AA Change** | **MutFunc** |
| --- | --- | --- | --- | --- | --- | --- |
| ARHGAP35  ARHGAP35 | NM_004491 | g.chr19:47423431delC | exon1 | c.1499delC | p.S500X | stopgain |
|  | NM_004491 | g.chr19:47424544G>C | exon1 | c.G2612C | p.R871P | missense |
| ARID1A | NM_006015 | g.chr1:27100183C>T | exon16 | c.C3979T | p.Q1327X | stopgain |
| ARID1A | NM_006015 | g.chr1:27107207delA | exon20 | c.6818delA | p.Q2273fs | frameshift deletion |
| ARID1A | NM_006015 | g.chr1:27023948delG | exon1 | c.1054delG | p.G352fs | frameshift deletion |
| ARID1A | NM_006015 | g.chr1:27107012-27107013insC | exon20 | c.6624dupC | p.F2208fs | frameshift insertion |
| ARID1A | NM_006015 | g.chr1:27105978-27105979delAG | exon20 | c.5589_5590del | p.T1863fs | frameshift deletion |
| ARID1A | NM_006015 | g.chr1:27101099C>T | exon18 | c.C4381T | p.R1461X | stopgain |
| CTNNB1  CTNNB1  CTNNB1 | NM_001904 | g.chr3:41266112T>C | exon3 | c.T109C | p.S37P | missense |
|  | NM_001904 | g.chr3:41266097G>A | exon3 | c.G94A | p.D32N | missense |
|  | NM_001904 | g.chr3:41266103G>A | exon3 | c.G100A | p.G34R | missense |
| PIK3CA  PIK3CA  PIK3CA  PIK3CA  PIK3CA  PIK3CA  PIK3CA  PIK3CA | NM_006218 | g.chr3:178947827G>T | exon19 | c.G2702T | p.C901F | missense |
|  | NM_006218 | g.chr3:178952085A>G | exon21 | c.A3140G | p.H1047R | missense |
|  | NM_006218 | g.chr3:178952018A>G | exon21 | c.A3073G | p.T1025A | missense |
|  | NM_006218 | g.chr3:178916930G>T | exon2 | c.G317T | p.G106V | missense |
|  | NM_006218 | g.chr3:178952072A>G | exon21 | c.A3127G | p.M1043V | missense |
|  | NM_006218 | g.chr3:178952085A>T | exon21 | c.A3140T | p.H1047L | missense |
|  | NM_006218 | g.chr3:178937393A>G | exon12 | c.A1781G | p.K594R | missense |
|  | NM_006218 | g.chr3:178936095A>G | exon10 | c.A1637G | p.Q546R | missense |
| PIK3R1 | NM_181523 | g.chr5:67589631-67589632insATTATATGAAGA | exon11 | c.1394_1395insATTATATGAAGA | p.R465delinsRLYEE | nonframeshift insertion |
| PIK3R1 | NM_181523 | g.chr5:67589639-67589640insAAGAATATACCC | exon11 | c.1402_1403insAAGAATATACCC | p.E468delinsEEYTQ | nonframeshift insertion |
| PTEN | NM_000314 | g.chr10:89717672C>T | exon7 | c.C697T | p.R233X | stopgain |
| PTEN | NM_000314 | g.chr10:89717699G>T | exon7 | c.G724T | p.E242X | stopgain |
| PTEN | NM_000314 | g.chr10:89692904C>G | exon5 | c.C388G | p.R130G | missense |
| PTEN | NM_000314 | g.chr10:89711927T>G | exon6 | c.T545G | p.L182X | stopgain |
| PTEN | . | g.chr10:89685315-89685318delGTAA | . | . | . | . |
| PTEN | NM_000314 | g.chr10:89692986delA | exon5 | c.470delA | p.E157fs | frameshift deletion |
| PTEN | NM_000314 | g.chr10:89624271-89624275delATATC | exon1 | c.45_49del | p.R15fs | frameshift deletion |
| SOX17 | NM_022454 | g.chr8:55370985C>G | exon1 | c.C287G | p.A96G | missense |
| SOX17 | NM_022454 | g.chr8:55372164-55372173delGCCTCCTGGC | exon2 | c.854_863del | p.G285fs | frameshift deletion |
| SOX17 | NM_022454 | g.chr8:55371731C>T | exon2 | c.C421T | p.R141W | missense |
| SPOP  SPOP | NM_003563 | g.chr17:47696640T>A | exon5 | c.A308T | p.K103I | missense |
|  | NM_003563 | g.chr17:47696599T>C | exon5 | c.A349G | p.M117V | missense |
| TP53 | NM_001126115 | g.chr17:7577599C>G | exon3 | c.G286C | p.D96H | missense |
| TP53 | NM_001126115 | g.chr17:7578263G>A | exon2 | c.C190T | p.R64X | stopgain |
| TP53 | NM_001126115 | g.chr17:7578195-7578197delCAC | exon2 | c.256_258del | p.86_86del | nonframeshift deletion |
| TP53 | NM_001126115 | g.chr17:7577124C>T | exon4 | c.G418A | p.V140M | missense |
| TP53 | NM_001126115 | g.chr17:7577557A>G | exon3 | c.T328C | p.C110R | missense |

**Table 3-B. OC gene-panel**

| **Gene** | **Transcript ID** | **Nucleotide (genomic)** | **Exon#** | **cDNA Change** | **AA Change** | **MutFunc** |
| --- | --- | --- | --- | --- | --- | --- |
| ARHGAP35 | NM_004491 | g.chr19:47422531-47422532insT | exon1 | c.600dupT | p.T200fs | frameshift insertion |
| ARID1A | NM_006015 | g.chr1:27100182-27100184delGCA | exon16 | c.3978_3980del | p.1326_1327del | nonframeshift deletion |
| ARID1A | NM_006015 | g.chr1:27094483-27094484delTG | exon11 | c.3191_3192del | p.L1064fs | frameshift deletion |
| ARID1A | NM_006015 | g.chr1:27106236delC | exon20 | c.5847delC | p.H1949fs | frameshift deletion |
| BRCA1 | NM_007300 | g.chr17:41276106A>T | exon2 | c.T8A | p.L3X | stopgain |
| BRCA1 | NM_007300 | g.chr17:41215920G>A | exon18 | c.C5186T | p.A1729V | missense |
| EGFR | NM_201284 | g.chr7:55220326G>C | exon6 | c.G716C | p.G239A | missense |
| EGFR | NM_005228 | g.chr7:55241738T>C | exon18 | c.2184+2T>C | . | . |
| LRRK2 | NM_198578 | g.chr12:40707952C>T | exon32 | c.C4715T | p.A1572V | missense |
| PIK3CA | NM_006218 | g.chr3:178936094C>A | exon10 | c.C1636A | p.Q546K | missense |
| PIK3CA | NM_006218 | g.chr3:178936095A>G | exon10 | c.A1637G | p.Q546R | missense |
| RAD21 | NM_006265 | g.chr8:117878960G>T | exon2 | c.C9A | p.Y3X | stopgain |
| TP53 | NM_001126115 | g.chr17:7577539G>A | exon3 | c.C346T | p.R116W | missense |
| TP53 | NM_001126118 | g.chr17:7579358C>G | exon3 | c.G212C | p.R71P | missense |
| TP53 | NM_001126115 | g.chr17:7578406C>T | exon1 | c.G128A | p.R43H | missense |
| TP53 | NM_001126115 | g.chr17:7577022G>A | exon4 | c.C520T | p.R174X | stopgain |
| TP53 | NM_001126115 | g.chr17:7578190T>C | exon2 | c.A263G | p.Y88C | missense |
| TP53 | NM_001126115 | g.chr17:7578212G>A | exon2 | c.C241T | p.R81X | stopgain |
| TP53 | NM_001126115 | g.chr17:7578386-7578387insC | exon1 | c.147_148insG | p.C50fs | frameshift insertion |
| TP53 | NM_001126115 | g.chr17:7578388-7578389insA | exon1 | c.145_146insT | p.R49fs | frameshift insertion |
| TP53 | NM_001126115 | g.chr17:7578527-7578528delAA | exon1 | c.6_7del | p.F2fs | frameshift deletion |
| TP53 | NM_001126115 | g.chr17:7576910delG | exon5 | c.540delC | p.T180fs | frameshift deletion |
| TP53 | NM_001126115 | g.chr17:7577556C>A | exon3 | c.G329T | p.C110F | missense |
| TP53 | NM_001126115 | g.chr17:7577120C>T | exon4 | c.G422A | p.R141H | missense |
| TP53 | NM_001126115 | g.chr17:7578190T>G | exon2 | c.A263C | p.Y88S | missense |
| TP53 | NM_001126118 | g.chr17:7579528C>T | exon3 | c.G42A | p.W14X | stopgain |
| TP53 | NM_001126115 | g.chr17:7577105delG | exon4 | c.437delC | p.P146fs | frameshift deletion |
| TP53 | NM_001126115 | g.chr17:7578265delA | exon2 | c.188delT | p.I63fs | frameshift deletion |
| TP53 | NM_001126115 | g.chr17:7578500G>A | exon1 | c.C34T | p.Q12X | stopgain |

**Table 4. Quality control of the sequencing data of tumor tissues and leukocytes**

| **Sample** | **Sample Type** | **Reads On Target(Mb)** | **Depth On Target** | **Fraction Target ≥ 1×** | **Fraction Target ≥ 10×** | **Fraction Target ≥ 50×** |
| --- | --- | --- | --- | --- | --- | --- |
| R0703EST1_319A | Tumor | 280.66 | 340.27 | 0.9991 | 0.9969 | 0.9884 |
| R0703EST2_320A | Tumor | 334.66 | 405.74 | 0.9993 | 0.9975 | 0.991 |
| R0703EST3_305A | Tumor | 242.73 | 294.29 | 0.9987 | 0.9957 | 0.9851 |
| R0703EST4_306A | Tumor | 248.51 | 301.29 | 0.999 | 0.9963 | 0.9863 |
| REST007A1 | Tumor | 451.66 | 547.6 | 0.9996 | 0.9977 | 0.9912 |
| REST008A1 | Tumor | 424.09 | 514.17 | 0.9991 | 0.9972 | 0.9913 |
| REST010A1 | Tumor | 412.47 | 500.08 | 0.9996 | 0.9976 | 0.9908 |
| REST011A1 | Tumor | 456.29 | 553.21 | 0.9997 | 0.9978 | 0.9915 |
| REST013A1 | Tumor | 296.06 | 358.95 | 0.999 | 0.9963 | 0.9864 |
| REST014A1 | Tumor | 309.07 | 374.72 | 0.9988 | 0.9963 | 0.9888 |
| REST017A1 | Tumor | 430.99 | 522.54 | 0.9994 | 0.9974 | 0.9925 |
| REST019A1 | Tumor | 472.8 | 573.23 | 0.9994 | 0.9977 | 0.9928 |
| REST020A1 | Tumor | 365.66 | 443.33 | 0.999 | 0.9965 | 0.9893 |
| RECT00201T | Tumor | 1068.21 | 1295.11 | 1 | 0.9997 | 0.9988 |
| RECT00401T | Tumor | 987.63 | 1197.41 | 0.9999 | 0.9994 | 0.9983 |
| RECT00501T | Tumor | 1033.03 | 1252.45 | 1 | 0.9994 | 0.9984 |
| RECT00701T | Tumor | 942.47 | 1142.66 | 0.9999 | 0.9994 | 0.9981 |
| RECT00801T | Tumor | 989.37 | 1199.52 | 1 | 0.9994 | 0.9981 |
| RECT01001T | Tumor | 979.2 | 1187.19 | 1 | 0.9994 | 0.9982 |
| RECT01201T | Tumor | 995.12 | 1206.5 | 0.9999 | 0.9993 | 0.9982 |
| RECT01301T | Tumor | 1131.14 | 1371.4 | 1 | 0.9996 | 0.9986 |
| RECT01501T | Tumor | 1096.97 | 1329.98 | 1 | 0.9997 | 0.9987 |
| R0703OST2_321A | Tumor | 218 | 264.31 | 0.9991 | 0.9964 | 0.9851 |
| R0703OST3 | Tumor | 245.59 | 297.76 | 0.9991 | 0.9963 | 0.9844 |
| R0703OST4 | Tumor | 226.87 | 275.06 | 0.999 | 0.9965 | 0.9858 |
| R0703OST5 | Tumor | 213.55 | 258.92 | 0.9989 | 0.9962 | 0.9842 |
| R0703OST6 | Tumor | 214.05 | 259.52 | 0.999 | 0.9964 | 0.9859 |
| ROST007A1 | Tumor | 449.91 | 545.47 | 0.9994 | 0.9974 | 0.9905 |
| ROST009A1 | Tumor | 409.32 | 496.26 | 0.9993 | 0.997 | 0.9904 |
| ROST010A1 | Tumor | 406.86 | 493.28 | 0.9991 | 0.9972 | 0.9915 |
| ROST011A1 | Tumor | 464.53 | 563.2 | 0.9994 | 0.9973 | 0.992 |
| ROST012A1 | Tumor | 424.46 | 514.62 | 0.9994 | 0.9975 | 0.9924 |
| ROST013A1 | Tumor | 346.68 | 420.32 | 0.9989 | 0.9967 | 0.9911 |
| ROST016A1 | Tumor | 355.22 | 430.67 | 0.9992 | 0.9967 | 0.988 |
| ROST017A1 | Tumor | 404.8 | 490.78 | 0.9994 | 0.9975 | 0.9922 |
| ROST019A1 | Tumor | 378.05 | 458.35 | 0.9987 | 0.9969 | 0.9907 |
| ROST020A1_325A | Tumor | 384.3 | 465.93 | 0.9993 | 0.9971 | 0.9916 |
| ROST02101T | Tumor | 1051.02 | 1274.27 | 1 | 0.9995 | 0.9983 |
| ROST02201T | Tumor | 1108.27 | 1343.67 | 1 | 0.9996 | 0.9985 |
| R0703ESB1_317A | Blood | 66.56 | 80.7 | 0.9978 | 0.9887 | 0.8743 |
| R0703ESB3_308A | Blood | 64.3 | 77.96 | 0.9974 | 0.9866 | 0.8514 |
| R0703ESB4_309A | Blood | 69.05 | 83.72 | 0.9977 | 0.9865 | 0.8856 |
| R0721XH29 | Blood | 76.44 | 92.68 | 0.9977 | 0.9898 | 0.8933 |
| RESB00201B | Blood | 130.18 | 157.83 | 0.9993 | 0.996 | 0.9798 |
| RESB008n2 | Blood | 160.95 | 195.13 | 0.9986 | 0.9953 | 0.9769 |
| RESB010n2 | Blood | 213.68 | 259.06 | 0.9989 | 0.9966 | 0.985 |
| RESB011n2 | Blood | 263.76 | 319.79 | 0.9995 | 0.9975 | 0.9855 |
| RESB013n2 | Blood | 90.4 | 109.6 | 0.9991 | 0.9914 | 0.9364 |
| RESB014n2 | Blood | 300.24 | 364.01 | 0.9994 | 0.9976 | 0.9928 |
| RESB017n2 | Blood | 234.19 | 283.93 | 0.9991 | 0.9969 | 0.9902 |
| RESB019n2 | Blood | 260.43 | 315.74 | 0.9996 | 0.9975 | 0.9914 |
| RESB020n2 | Blood | 261.62 | 317.19 | 0.9993 | 0.9974 | 0.9918 |
| RECB00201N | Blood | 416.48 | 504.94 | 0.9997 | 0.999 | 0.9953 |
| RECB00401N | Blood | 451.92 | 547.92 | 0.9999 | 0.9991 | 0.9957 |
| RECB00501N | Blood | 443.27 | 537.42 | 0.9999 | 0.9991 | 0.9953 |
| RECB00701N | Blood | 374.07 | 453.53 | 0.9998 | 0.9988 | 0.9944 |
| RECB00801N | Blood | 441.97 | 535.84 | 0.9997 | 0.9991 | 0.9956 |
| RECB01001N | Blood | 400.08 | 485.06 | 1 | 0.999 | 0.9949 |
| RECB01201N | Blood | 365.58 | 443.23 | 0.9999 | 0.9988 | 0.9944 |
| RECB01301N | Blood | 342.73 | 415.53 | 0.9998 | 0.9987 | 0.994 |
| RECB01501N | Blood | 322.83 | 391.4 | 0.9997 | 0.9986 | 0.9936 |
| R0703OSB2_312A | Blood | 85.09 | 103.16 | 0.9979 | 0.9913 | 0.9338 |
| R0703OSB3_313A | Blood | 87.61 | 106.23 | 0.9982 | 0.9903 | 0.9376 |
| R0703OSB4_314A | Blood | 60.49 | 73.33 | 0.9976 | 0.9884 | 0.8191 |
| R0703OSB5_315A | Blood | 72.38 | 87.75 | 0.9976 | 0.9881 | 0.8916 |
| R0703OSB6_316A | Blood | 84.28 | 102.18 | 0.9987 | 0.9923 | 0.9259 |
| R0721XH27 | Blood | 74.52 | 90.35 | 0.9972 | 0.9891 | 0.8783 |
| ROSB00901B | Blood | 207.8 | 251.94 | 0.9993 | 0.9961 | 0.9875 |
| ROSB010n2 | Blood | 260.29 | 315.58 | 0.9993 | 0.9971 | 0.9909 |
| ROSB011n2 | Blood | 316.02 | 383.15 | 0.9993 | 0.9976 | 0.9927 |
| ROSB012n2 | Blood | 265.56 | 321.97 | 0.9993 | 0.997 | 0.9912 |
| ROSB013n2 | Blood | 312.65 | 379.06 | 0.9992 | 0.9973 | 0.9926 |
| ROSB01601 | Blood | 397.32 | 481.71 | 0.9992 | 0.998 | 0.9939 |
| ROSB01701 | Blood | 358.63 | 434.81 | 0.9995 | 0.9979 | 0.9933 |
| ROSB01901 | Blood | 283.65 | 343.9 | 0.9992 | 0.9974 | 0.9916 |
| ROSB02001 | Blood | 394.95 | 478.84 | 0.9991 | 0.998 | 0.9937 |
| ROSB02101N | Blood | 313.75 | 380.4 | 0.9999 | 0.9989 | 0.9933 |
| ROSB02201N | Blood | 284.04 | 344.37 | 0.9998 | 0.9984 | 0.9928 |

**Table 5 TP53 mutations and CTNNB1 mutations in EC tumors**

A. The TP53 somatic mutations in Poor group

| **SampleID** | **GeneSymbol** | **Exon#** | **cDNA_Change** | **AA_Change** | **Mutation Function** |
| --- | --- | --- | --- | --- | --- |
| REST007A1 | *TP53* | exon2 | c.C190T | p.R64X | stopgain |
| REST008A1 | *TP53* | exon2 | c.256_258del | p.86_86del | nonframeshift deletion |
| REST010A1 | *TP53* | exon4 | c.G418A | p.V140M | nonsynonymous SNV |
| REST011A1 | *TP53* | exon3 | c.T328C | p.C110R | nonsynonymous SNV |
| RECT00701T | *TP53* | exon8 | c.783-2A>C | . | alternate splicing |

B. The TP53 somatic mutations in Good group

| **SampleID** | **GeneSymbol** | **Exon#** | **cDNA_Change** | **AA_Change** | **Mutation Function** |
| --- | --- | --- | --- | --- | --- |
| R0703EST1_319A | *TP53* | exon3 | c.G286C | p.D96H | nonsynonymous SNV |
|  | *TP53* | exon4 | c.G443C | p.R148T | nonsynonymous SNV |

C. The CTNNB1 somatic mutations in Good group

| **SampleID** | **GeneSymbol** | **Exon#** | **cDNA Change** | **AA Change** | **Mutation Function** | **Effect** |
| --- | --- | --- | --- | --- | --- | --- |
| R0703EST3_305A | *CTNNB1* | exon3 | c.T109C | p.S37P | nonsynonymous SNV | GOF* |
| R0703EST4_306A | *CTNNB1* | exon3 | c.G94A | p.D32N | nonsynonymous SNV | GOF |
| REST017A1 | *CTNNB1* | exon3 | c.G100A | p.G34R | nonsynonymous SNV | GOF |
| RECT00201T | *CTNNB1* | exon3 | c.A95C | p.D32A | nonsynonymous SNV | GOF |
| RECT00801T | *CTNNB1* | exon3 | c.T109C | p.S37P | nonsynonymous SNV | GOF |
| RECT01001T | *CTNNB1* | exon3 | c.C110T | p.S37F | nonsynonymous SNV | GOF |
| RECT01201T | *CTNNB1* | exon3 | c.C110T | p.S37F | nonsynonymous SNV | GOF |
| RECT01501T | *CTNNB1* | exon3 | c.C110G | p.S37C | nonsynonymous SNV | GOF |

*GOF: gain of function (information from JAX-Clinical Knowledgebase)
